# A DUF-246 family glycosyltransferase-like gene affects male fertility and the biosynthesis of pectic arabinogalactans

**DOI:** 10.1186/s12870-016-0780-x

**Published:** 2016-04-18

**Authors:** Solomon Stonebloom, Berit Ebert, Guangyan Xiong, Sivakumar Pattathil, Devon Birdseye, Jeemeng Lao, Markus Pauly, Michael G. Hahn, Joshua L. Heazlewood, Henrik Vibe Scheller

**Affiliations:** Joint BioEnergy Institute and Biological Systems and Engineering, Lawrence Berkeley National Laboratory, Berkeley, CA 94720 USA; Department of Plant and Environmental Sciences, Faculty of Science, University of Copenhagen, C 1871 Copenhagen, Denmark; Energy Biosciences Institute, University of California, Berkeley, CA 94720 USA; Department of Plant and Microbial Biology, University of California, Berkeley, CA 94720 USA; Complex Carbohydrate Research Center, University of Georgia, Athens, GA 30602-4712 USA; BioEnergy Science Center, University of Georgia, Athens, GA 30602-4712 USA; Department of Plant Biology, University of Georgia, Athens, GA 30602-4712 USA; ARC Centre of Excellence in Plant Cell Walls, School of Botany, The University of Melbourne, 3010 Melbourne, Victoria Australia

**Keywords:** *Arabidopsis thaliana*, *Nicotiana benthamiana*, Cell wall, Rhamnogalacturonan-I, Pectin, Pollen tube

## Abstract

**Background:**

Pectins are a group of structurally complex plant cell wall polysaccharides whose biosynthesis and function remain poorly understood. The pectic polysaccharide rhamnogalacturonan-I (RG-I) has two types of arabinogalactan side chains, type-I and type-II arabinogalactans. To date few enzymes involved in the biosynthesis of pectin have been described. Here we report the identification of a highly conserved putative glycosyltransferase encoding gene, *Pectic ArabinoGalactan synthesis-Related* (*PAGR*), affecting the biosynthesis of RG-I arabinogalactans and critical for pollen tube growth.

**Results:**

T-DNA insertions in *PAGR* were identified in *Arabidopsis thaliana* and were found to segregate at a 1:1 ratio of heterozygotes to wild type. We were unable to isolate homozygous *pagr* mutants as *pagr* mutant alleles were not transmitted via pollen. *In vitro* pollen germination assays revealed reduced rates of pollen tube formation in pollen from *pagr* heterozygotes. To characterize a loss-of-function phenotype for *PAGR,* the *Nicotiana benthamiana* orthologs, *NbPAGR-A* and *B,* were transiently silenced using Virus Induced Gene Silencing. *NbPAGR-*silenced plants exhibited reduced internode and petiole expansion. Cell wall materials from *NbPAGR*-silenced plants had reduced galactose content compared to the control. Immunological and linkage analyses support that RG-I has reduced type-I arabinogalactan content and reduced branching of the RG-I backbone in *NbPAGR-*silenced plants. Arabidopsis lines overexpressing PAGR exhibit pleiotropic developmental phenotypes and the loss of apical dominance as well as an increase in RG-I type-II arabinogalactan content.

**Conclusions:**

Together, results support a function for *PAGR* in the biosynthesis of RG-I arabinogalactans and illustrate the essential roles of these polysaccharides in vegetative and reproductive plant growth.

**Electronic supplementary material:**

The online version of this article (doi:10.1186/s12870-016-0780-x) contains supplementary material, which is available to authorized users.

## Background

**Fig. 1:**
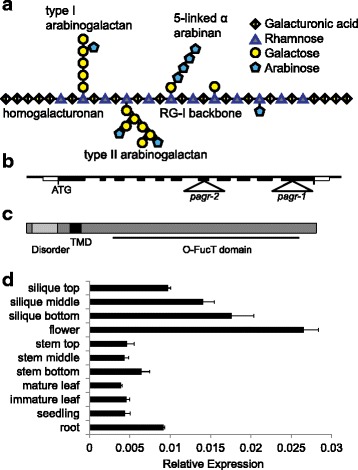
Schematic representation of the pectic polysaccharide rhamnogalacturonan-I (**a**) and the structure and expression of *PAGR* (**b**-**d**)*.* **a** Rhamnogalacturonan-I has a backbone consisting of repeating [−α-d-Gal*p*A-1,2-α-l-Rha*p*-1,4-] disaccharide units. Approximately half of the rhamnose residues are substituted with sidechains on the 4-position such as type-I and type-II arabinogalactans, α-(1,5)-linked arabinans or single arabinose or galactose residues. **b** *pagr-1* is a T-DNA insertion in the 11^th^ exon of *PAGR; pagr-2* is an insertion in the 7^th^ exon. **c** *PAGR* encodes a type-II membrane protein with an N-terminal disordered domain and a C-terminal *O*-fucosyltransferase-like DUF246 domain. **d** Quantitative RT-PCR analysis of *PAGR* expression in various Arabidopsis tissues. Results show *PAGR* expression is highest in reproductive tissues and roots. Error bars indicate standard deviation, *n* = 3

Pectins are an important group of structural cell wall polysaccharides in plants and are major constituents of primary cell walls. Pectins are also important components of many foods and food products. Along with the hemicelluloses, pectins form a matrix into which cellulose microfibrils are embedded. Pectins comprise several types of acidic polysaccharide domains: homogalacturonan, xylogalacturonan, rhamnogalacturonan-I (RG-I) and rhamnogalacturonan-II (RG-II), that may be interconnected to one another as well as to other polysaccharides and glycoproteins including hemicelluloses and arabinogalactan proteins (AGPs) [1, 2]. Homogalacturonan consists of linear chains of **α**-1-4-linked d-galacturonic acid residues and can account for 60 % or more of pectin. Galacturonic acid residues are methyl esterified to varying degrees and can be acetylated at O-2 or O-3 in both homogalacturonan and RG-I. In some plants or tissues homogalacturonan is substituted with xylose or apiose making xylogalacturonan and apiogalacturonan, respectively. RG-I (diagrammed in Fig. 1a) is a distinct class of pectic polysaccharides with a backbone consisting of repeating [−α-d-Gal*p*A-1,2-α-l-Rha*p*-1,4-] disaccharide units [3]. Approximately half of the rhamnose residues are elaborated with sidechains on the 4-position, including β-1,4-linked d-Gal*p* chains, α-(1,5)-linked l-Ara*f* chains as well as type I and II–arabinogalactans. Type-I arabinogalactans consist of β-1,4-linked d-Gal*p* backbones with arabinan sidechains, while type-II arabinogalactans possess a backbone of β-1,3-, β-1,6-and β-1,3,6- linked d-Gal*p* similar to the arabinogalactans of AGPs. Rhamnogalacturonan-II is a form of homogalacturonan substituted with four characteristic types of elaborate side chains with distinctive structures made up of 13 different types of monosaccharides and containing the rare sugars l-Aceric acid, d-Apiose, 2-keto-3-deoxy-d-Lyxo-heptulosaric acid (Dha) and 2-keto-3-deoxy-d-Manno-octulosonic acid (Kdo) [4, 5]. RG-II forms dimers through boron di-ester bonds and is a ubiquitous component of plant cell walls, highlighting its importance. Given its structural complexity, as many as 67 different transferase activities are thought to be necessary for the biosynthesis of pectin [1].

Plant cell wall polysaccharides are mainly synthesized by glycosyltransferases, enzymes that transfer monosaccharides from an activated donor substrate, usually a nucleotide sugar, onto acceptor molecules, forming glycosidic bonds. Most glycosyltransferases exhibit strong selectivity for donor sugar and acceptor substrates [6]. Glycosyltransferases are classified as “inverting” or “retaining” enzymes depending on whether glycosylation occurs with retention or inversion of stereochemistry at the anomeric carbon atom of the donor substrate. These enzymes have been further classified into families on the basis of amino acid sequence similarities in the Carbohydrate Active enZyme database (CAZy) [7]. The Arabidopsis genome contains 463 genes classified into 41 distinct glycosyltransferase gene families and an additional 100 glycosyltransferase-like genes possessing insufficient similarity to characterized enzymes to be assigned to a CAZy gene family.

To date, only a few enzymes involved in the biosynthesis of pectin have been characterized. These include GAUT1, which is a homogalacturonan galacturonosyltransferase in glycosyltransferase (GT) family 8 [8]. Another GT family 8 member, GATL5, has been shown to be involved in the synthesis of *Arabidopsis* seed coat mucilage, which is largely composed of RG-I, though its precise role in mucilage biosynthesis has not been established [9]. Xylosyltransferases found in GT family 77, RhamnoGalacturonan-II XylosylTransferase-1 (RGXT1), RGXT2 and RGXT3, have been shown to xylosylate l-fucose in the A-chain of RG-II [10, 11]. ARAD1 and 2 belong to GT family 47 and are involved in the biosynthesis of arabinans on RG-I; however their catalytic activity has not been demonstrated [12, 13]. XGD1 also belongs to GT family 47 and is a xylosyltransferase that adds xylose to homogalacturonan to form xylogalacturonan [14]. A recently characterized galactan synthase, GALS1, in GT family 92 extends pectic β-1,4-galactan [15]. GALS1 requires at least a galactotetraose oligosaccharide as a substrate indicating that it extends but does not initiate RG-I galactan biosynthesis. Thus, additional unidentified enzymes are apparently required for the initial branching of RG-I. A recent study suggests that at least some RG-I may be produced as a proteoglycan attached to AGP-polysaccharides [16].

The structure and composition of pectins are altered during growth, development and in response to changing conditions. RG-I has specific roles in many plant organs and tissues, and is present in all primary plant cell walls [17]. RG-I is likely present in the cell walls of all vascular plants and has been detected in the walls of basal land plants such as *Physcomitrella patens* as well as the inner cell wall of the Charophyte alga *Penium magaritaceum* [18]. Homogalacturonan plays a critical role in the walls of tip growing cells such as root hairs and pollen tubes where de-methyl esterification and cross-linking of homogalacturonan at the edge of the growing tip is thought to solidify the nascent cell wall [19]. RG-I arabinogalactans are also critical components of the pollen tube cell wall. In olive plants pectic galactan forms a ring around the aperture where pollen tubes emerge from the pollen grain [20]. Pectic arabinans likely present as sidechains on RG-I are a critical part of the pollen cell wall [21].

A better understanding of the biosynthesis and processing of pectins is critical to elucidating how this enigmatic class of polymers functions in regulating properties of the plant cell wall. Many uncharacterized GT activities are required for the biosynthesis of pectic polysaccharides. Here we report the identification of a new gene affecting pollen tube growth and present evidence that it is involved in the biosynthesis of pectic arabinogalactans attached to RG-I. We have named the Arabidopsis gene, *At3g26370*, *Pectic ArabinoGalactan synthesis-Related (PAGR)*.

## Results

### PAGR is a highly conserved DUF-246 domain containing protein

Land plants have an expanded group of genes encoding proteins related to GT family 65, the DUF246 family of GT65-like proteins with 39 members in Arabidopsis [22]. The mammalian GT family 65 protein POFUT1 fucosylates serine/threonine in epidermal growth factor repeats [23]. As the genes that contain a DUF246 domain are an expanded family in plants, some of them are likely to be involved in plant-specific processes [22]. The only previously studied plant DUF246-encoding genes are *MSR1* and *MSR2*, which affect the production and secretion of mannans in Arabidopsis; however their specific role in mannan biosynthesis remains elusive [24]. In examining the predicted amino acid sequences of plant DUF246-containing proteins we identified a gene, *At3g26370* (*PAGR*), encoding a protein that is more highly conserved throughout the land plants than other DUF246 containing proteins. The *Selaginella moellendorffii* and *Physcomitrella patens* orthologs of PAGR are 76.0 % and 70.9 % identical to the Arabidopsis protein, respectively (Additional file 1: Figure S1). Other Arabidopsis DUF246 proteins have basal land plant orthologs with pairwise amino acid identities between 45 and 67.7 %. The strong conservation of PAGR throughout the land plants can be observed as the short branch length within the PAGR clade in phylogenetic analysis of the DUF246 containing proteins in Arabidopsis, *Selaginella* and *Physcomitrella* (Additional file 2: Figure S2). *PAGR* is predicted to encode a type-II membrane protein with an N-terminal disordered domain, a transmembrane domain and the highly conserved DUF246 domain (Fig. 1c).

### *PAGR* is expressed ubiquitously

We examined the expression pattern of *PAGR* in Arabidopsis using quantitative RT-PCR of RNA prepared from various tissues (Fig. 1d). *PAGR* was expressed in all tissues tested with higher levels of transcript detected in reproductive tissues and roots. Transcript levels varied less than 7-fold between the tissues with the highest (flowers) and lowest (mature leaves) expression. This result is in agreement with publicly available microarray data showing expression of *PAGR* throughout the plant (Additional file 3: Figure S3) [25].

### *PAGR* mutant alleles are not transmitted via pollen

Two independent T-DNA insertions were identified in the coding sequence of *PAGR*, *pagr-1* in the eleventh exon and *pagr-2* in the seventh exon (Fig. 1b). We failed to isolate homozygous mutant seedlings in the progeny of plants heterozygous for either mutant allele. *pagr-1* and *pagr-2* mutant alleles were inherited by 41 % and 44 % of the progeny of heterozygous parents, indicating that the *pagr* mutant alleles were not transmitted by the male or female gametophyte (Table 1). χ^2^ tests of these segregation ratios support that both *pagr* mutant alleles are inherited in a 0:1:1 ratio of homozygous mutants to heterozygotes to wild type as is expected if mutant alleles are not transmitted by either the male or female gametophyte. We then performed reciprocal crosses of *pagr-1* and *pagr-2* heterozygotes and the wild type to test the transmission of *pagr* mutant alleles. In crosses where wild-type stigmas were fertilized with pollen from *pagr-1* or *pagr-*2 heterozygotes, *pagr* mutant alleles were not detected in the F1 progeny (Table 2). When *pagr-1* or *pagr-*2 heterozygotes were fertilized with wild-type pollen, mutant alleles were inherited by approximately 50 % of the F1 progeny. χ^2^ tests reject the hypothesis that *pagr* mutant alleles were transmitted via pollen but accept the hypothesis that mutant alleles were transmitted at the expected 1:1 ratio by female gametophytes. Thus, *pagr* mutant alleles were not transmitted by pollen but were transmitted normally by the female gametophyte.

**Table 1:** Segregation ratio of *PAGR* mutant alleles. The genotype of offspring from selfed heterozygotic *pagr* mutants was determined by PCR. The observed segregation ratios are consistent with a 1:1 segregation as expected with non-functional pollen

Line:	pagr/ pagr	pagr/ PAGR	PAGR/ PAGR	Proportion	p-value, χ2 test of 1:2:1 segregation	p-value, χ2 test of 0:1:1 segregation
pagr-1	0	31	45	0.41	7.4E-13	0.11
pagr-2	0	34	43	0.44	2.2E-11	0.31

**Table 2:** Segregation of *pagr* mutant alleles in the progeny of reciprocal crosses between *pagr* heterozygotes and wild type

Cross	pagr/PAGR	PAGR/PAGR	Proportion	p-value, χ2 test of 1:1 segregation
Col ♀ x *pagr-1/PAGR* ♂	0	94	0.00	3.16E-22
*pagr-1/PAGR* ♀ x Col ♂	34	42	0.81	0.36
Col ♀ x *pagr-2/PAGR* ♂	0	94	0.00	3.16E-22
*pagr-2/PAGR* ♀ x Col ♂	47	46	1.02	0.92

**Fig. 2:**
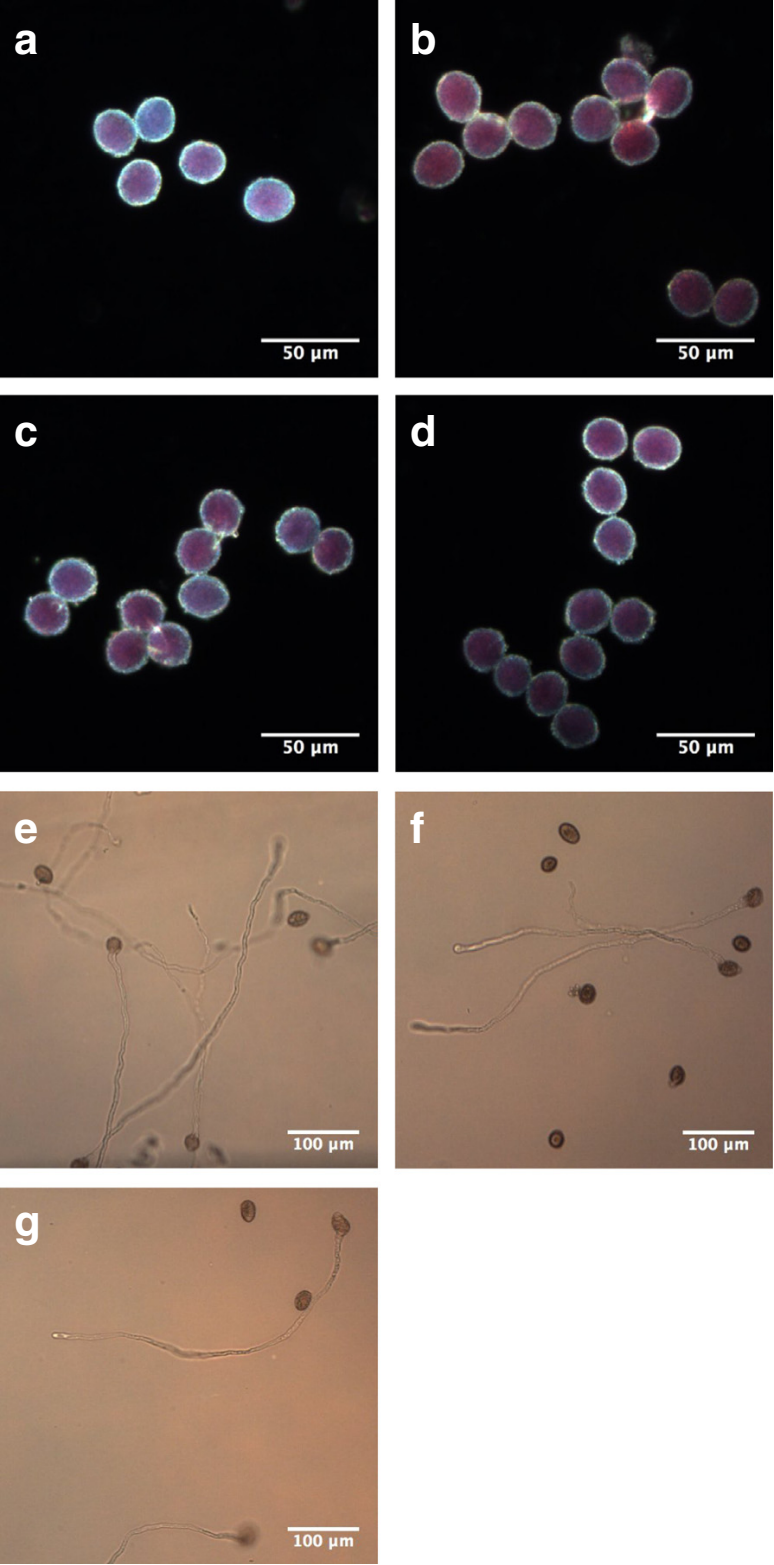
Pollen phenotypes of *pagr* heterozygotes. Pollen from segregating *PAGR/PAGR* (**a**), *pagr-1/PAGR* (**b** *), PAGR/PAGR* (**c**) and *pagr-2/PAGR* (**d**) plants stained equally with Alexander’s stain indicating that *pagr* pollen is viable. Scale bars 50 μm. In vitro pollen germination assays show reduced germination rates compared to the wild type (**e**) for pollen from *pagr-1/PAGR* (**f**) and *pagr-2/PAGR* (**g**) plants. Scale bars 100 μm

We therefore tested for phenotypes affecting pollen in *pagr* heterozygotes. The viability of pollen grains was tested with Alexander’s stain in segregating wild-type and *pagr* heterozygous plants [26]. More than 95 % of pollen grains from *pagr-1/PAGR, pagr-2/PAGR* and *PAGR/PAGR* plants stained purple with Alexander’s stain (Fig. 2a-d) indicating that development proceeds normally in *pagr* pollen prior to anthesis.

Pollen from wild type, and from *pagr-1/PAGR* and *pagr-2/PAGR* heterozygotes was germinated *in vitro* to determine if pollen germination or pollen tube growth are affected by PAGR. We observed an approximate 50 % decrease in the proportion of pollen grains producing pollen tubes in vitro from *pagr-1* and *pagr-2* heterozygotes (Table 3). This suggests that *pagr* pollen failed to germinate or to produce pollen tubes in vitro. There were no apparent alterations in the morphology of pollen tubes produced by pollen from *pagr* heterozygous plants (Fig. 2 e-g & Additional file 4: Figure S4). We thus concluded that mutation of *PAGR* disrupts pollen germination but does not affect the development of pollen prior to germination.

**Table 3:** Pollen tube growth analysis of *pagr* heterozygotes and the wild type

Pollen tube:	+	-	proportion of germinating pollen
wild type (Col-0)	229	95	0.71
*pagr-1/PAGR*	209	321	0.39*
*pagr-2/PAGR*	192	245	0.44*

Pollen was germinated in vitro and germination rates were scored following imaging. *: *p* < 0.001, population proportion Z-score test

### PAGR is localized to the Golgi apparatus and to small punctate structures

**Fig. 3:**
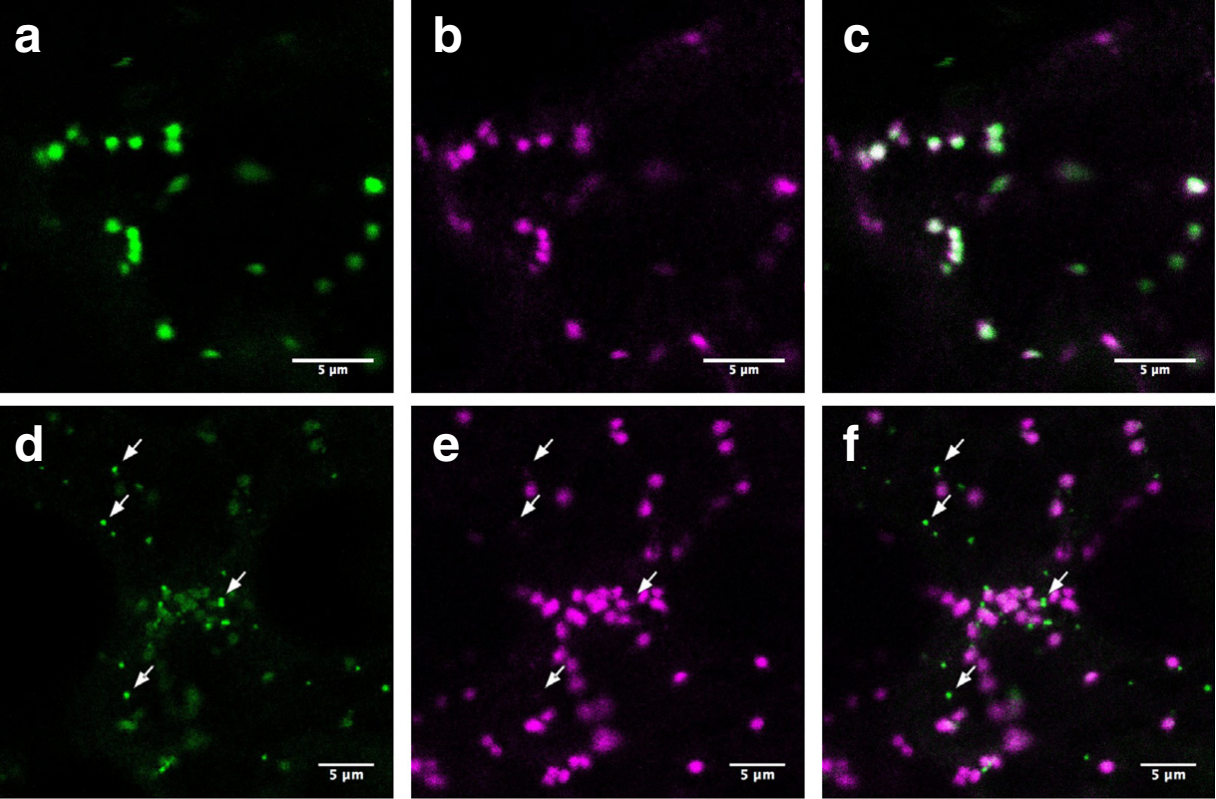
The subcellular localization of PAGR-CFP. PAGR-CFP (**a** & **d**) was co-expressed with the Golgi apparatus marker α-mannosidase-1-mCherry (**b** & **e**) in *N. benthamiana* leaves; merged signals (**c** & **f**). In some cells PAGR-CFP co-localized with α-mannosidase-1-mCherry (**a**-**c**). In most cells PAGR-CFP partially co-localized with α-mannosidase-1-mCherry and also localized to small punctate structures (white arrows, **d**-**f**). Scale bars 5 μm

The PAGR protein is predicted to be a type-II membrane protein (Fig. 1c) and has been detected by LC-MS/MS in the proteome of Golgi purified from Arabidopsis suspension cell cultures [27]. To confirm the localization of PAGR we transiently co-expressed PAGR-CFP in *N. benthamiana* with α-mannosidase-1 fused to mCherry as a Golgi marker [28]. In some cells PAGR co-localized with α-mannosidase-1 (Fig. 3a-c). However, in most cells PAGR-CFP also localized to small punctate structures in which α-mannosidase-1 was not present (Fig. 3d-f). This localization pattern to both the Golgi apparatus and to a smaller, non-Golgi subcellular compartment is similar to the localization of AtGALT31A, AtGALT29A and AtGlcAT14A [29]. AtGALT31A and AtGALT29A are galactosyltransferases involved in the biosynthesis of AGP type-II arabinogalactans [30, 31], and AtGlcAT14A is a glucuronosyltransferase also involved in the biosynthesis of type-II arabinogalactans [32]. Galt29A was reported to partially colocalize with EXO70E2 in small subcellular compartments thought to be involved in an unconventional secretion pathway known as Exocyst-Positive organelles (EXPOs) [29, 33]; however we have been unable to replicate this result.

### Silencing of *PAGR* in *Nicotiana benthamiana* affects pectin biosynthesis

**Fig. 4:**
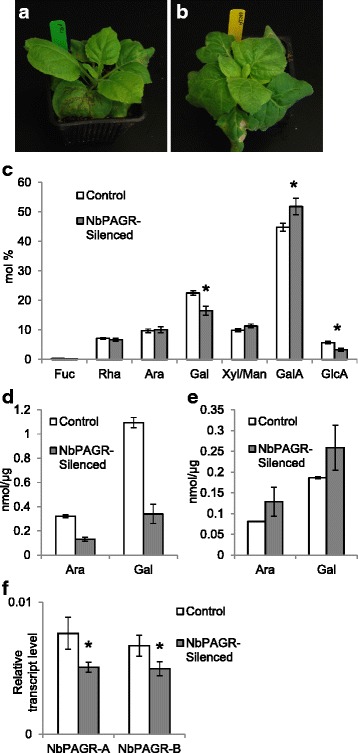
The phenotype of *NbPAGR-*silencing in *N.benthamiana*. **a**-**b** The morphological phenotype of control (**a**) and *NbPAGR*-silenced (**b**) plants. **c** The monosaccharide composition of control and *NbPAGR-*silenced cell walls. Values significantly different from the control are indicated with an asterisk (*n* = 5, t-test, *p* < 0.005). **d**-**e** β-1,4-galactanase treatment of NbPAGR and control cell walls. Solubilized sugars (**d**) and residual material (**e**) (*n* = 3). **f** Relative expression of *NbPAGR-A & NbPAGR-B* in silenced and control plants (*: *p* < 0.01, *n* = 4). Error bars indicate standard deviation

As we were unable to isolate homozygous Arabidopsis *pagr* mutants, we transiently silenced the *N. benthamiana* orthologs of *PAGR*, (*NbPAGR*) using Tobacco Rattle Virus-based Virus Induced Gene Silencing (VIGS) [34]. VIGS is a rapid and robust method for down-regulating genes of interest and has proven to be an effective system for studying plant cell wall formation and GT function [35, 36]. We identified two *PAGR* orthologs in the genome of *N. benthamiana, NbPAGR-A* and *NbPAGR-B* [37]. *NbPAGR-A* and *-B* encode proteins with 80.2 % and 80.6 % identity to Arabidopsis PAGR (Additional file 1: Figure S1) and phylogenetic analysis of these sequences shows strong homology to PAGR and its basal land plant orthologs (Additional file 5: Figure S5). *NbPAGR-A* and *–B* are 97.2 % identical to one another, sufficient similarity for efficient silencing of both orthologs with a single construct. A fragment of *NbPAGR-A* was cloned into a pTRV2 vector to induce silencing of both *N. benthamiana PAGR* orthologs*. N. benthamiana* plants were infected with TRV-VIGS viruses by infiltration with *Agrobacterium* containing *NbPAGR* and control TRV2-plasmids. *NbPAGR*-silenced plants had a stout phenotype resulting from cessation of internode and petiole expansion (Fig. 4). The leaves of *NbPAGR* silenced plants were tightly clustered around the shoot apical meristem. This phenotype is reminiscent of that resulting from silencing of UDP-Apiose/UDP-xylose synthase in *N. benthamiana,* which causes a deficiency in RG-II production [38]. We also observed alterations in root growth in *NbPAGR-*silenced plants, including decreased total root length and discoloration (Additional file 6: Figure S6). We evaluated the specificity and efficacy of gene silencing by performing real-time RT-PCR with primers specific for *NbPAGR-A* and *-B*. Levels of *NbPAGR-A* and *–B* transcripts were reduced by 34 % and 26 %, respectively in silenced plants (Fig. 4f). While this reduction in transcript levels is relatively minor, these results were consistent, statistically significant and may be explained by cessation of growth in tissues with significant silencing.

As *PAGR* was predicted to be involved in the production of plant cell wall polysaccharides, we analyzed the monosaccharide composition of cell wall material from *NbPAGR-*silenced and virus-infected non-silenced control plants. *NbPAGR*-silenced plants exhibited a significantly altered monosaccharide composition including a 33 % decrease in galactose content, a small decrease in glucuronic acid content, and increased galacturonic acid content (Fig. 4c). Sequential extraction of the cell wall material showed that less galactose and arabinose were present in pectic extracts obtained from *NbPAGR*-silenced cell walls with the chelating agent cyclohexane diamine tetraacetic acid (CDTA) and with sodium carbonate (Additional file 7: Figure S7). The cell wall material remaining after the extraction of pectic polysaccharides did not exhibit differences in composition between silenced and virus-infected control plants.

To determine if the observed changes in pectic galactose content were caused by a reduction in β-1,4-galactan, cell wall material was digested with endo-1,4-β-galactanase from *Aspergillius niger* and the solubilized and residual materials were subsequently analyzed. Treatment with the 1,4-β-galactanase released 68.9 % less galactose from cell walls of *NbPAGR-*silenced plants than from cell walls of the control plants (Fig. 4d-e), indicating that the reduction in total galactose content was related to a significant reduction in pectic galactan content and/or enzymatic accessibility. Interestingly, less arabinose was released following β-1,4 galactanase treatment from *NbPAGR-*silenced cell wall material as well, suggesting that type-I arabinogalactans were affected by *NbPAGR-* silencing in addition to β-1,4-galactan. Residual material following galactanase digestion was similar in composition in *NbPAGR*-silenced and control cell walls (Fig. 4e).

### Overexpression of *PAGR* in Arabidopsis causes ectopic phenotypes

**Fig. 5:**
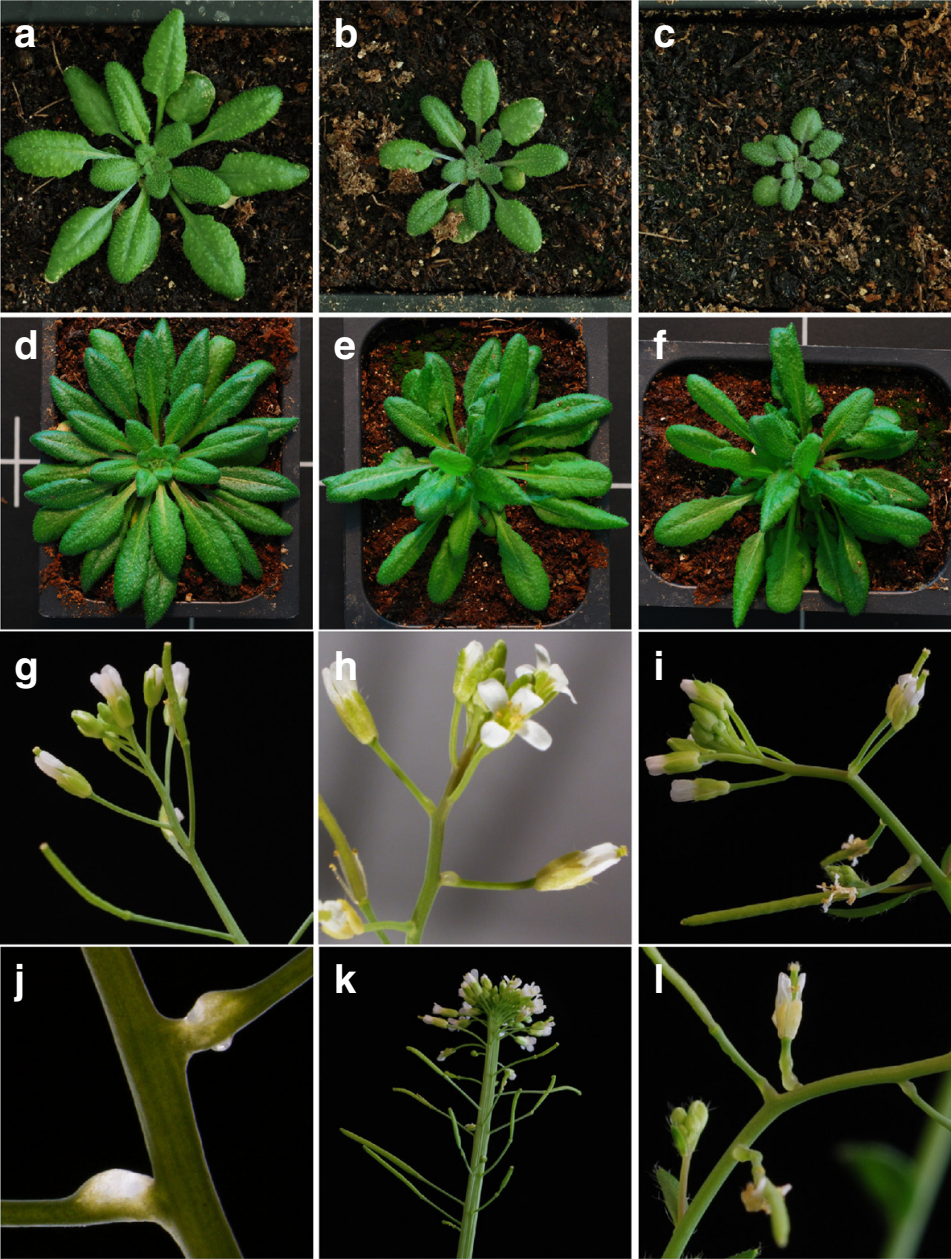
*PAGR* overexpression phenotypes in Arabidopsis rosettes. **a** 4-week old wild type, **b** *35S::PAGR-YFP* line 6, and **c** *35S::PAGR-YFP* line 9 plants. **d** 6-week old wild type, **e** *35S::PAGR-YFP* line 6 and **f** *35S::PAGR-YFP* line 9 plants. *35S::PAGR-YFP* inflorescences exhibit pleiotropic phenotypes. **g** Normal wild type inflorescence. **h** Inflorescence displaying swollen floral pedicels in *35S::PAGR-YFP* line 6. **i** Altered phyllotaxy and swollen pedicels in *35S::PAGR-YFP* line 9. **j** Close-up of swollen pedicel tissues in *35S::PAGR-YFP* line 6. **k** Fasciation in *35S::PAGR-YFP* line 6. **l** Swollen pedicels and altered patterning in *35S::PAGR-YFP* line 9

To investigate the effects of PAGR overexpression we expressed *PAGR-YFP* in Arabidopsis under the control of the cauliflower mosaic virus 35S promoter. Two independent 35S::PAGR-YFP lines were selected for further analysis. Seedlings overexpressing *PAGR* had a slightly dwarfed phenotype, with rosettes growing more slowly than the wild type (Fig. 5a-c). When grown under short-day conditions *PAGR* overexpressing plants lost apical dominance and developed multiple rosettes of tangled leaves (Fig. 5d-f). Under long-day conditions loss of apical dominance was observed infrequently. Inflorescences of 35S::PAGR-YFP plants had variable pleiotropic phenotypes each exhibited in independent transformants including fasciation, increased branching, swollen translucent floral pedicels and deficiencies in internode expansion (Fig. 5h-l).

**Fig. 6:**
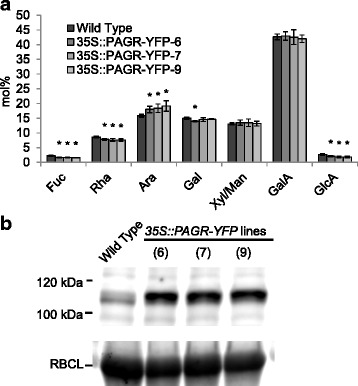
Biochemical characterization of PAGR overexpressing Arabidopsis lines. **a** The monosaccharide composition of cell walls from *35S::PAGR-YFP* seedlings grown in liquid culture. Over-expressing lines exhibit an increase in total arabinose content and slight reductions in fucose, rhamnose and glucuronic acid content. (*: *p* < 0.05, t-test, *n* = 4). **b** Expression analysis of PAGR-YFP in transgenic lines. Protein extracts from 10-day old T3 *35S::PAGR-YFP* seedlings and from the Wild Type were analyzed by immunoblotting and PAGR-YFP was detected with ‘universal antibody’ against the AttB2 site linker peptide in PAGR-YFP. Coomassie staining of Rubisco large subunit (RBCL) is shown as a loading control

Analysis of total cell wall material from three independent lines of 35S::PAGR-YFP plants showed a small increase in total arabinose content and small decreases in fucose, rhamnose and glucuronic acid content (Fig. 6a). Following sequential extraction of cell wall material from PAGR-overexpressing plants, we found that the CDTA cell wall extract contained an increased arabinose content whereas the subsequent 4 M KOH extract and the residual material showed no differences in monosaccharide composition compared to the wild type (Additional file 8: Figure S8). The expression of PAGR-YFP fusion protein in transgenic plants was confirmed by immunoblotting (Fig. 6b). PAGR-YFP was detected in all transgenic lines at a molecular mass of around 110 kDa, somewhat larger than the predicted molecular mass of 92.3 kDa, with antibody against the AttB2 linker peptide present in PAGR-YFP.

### *NbPAGR* silenced cell walls contain rhamnogalacturonan-I deficient in arabinogalactan

**Fig. 7:**
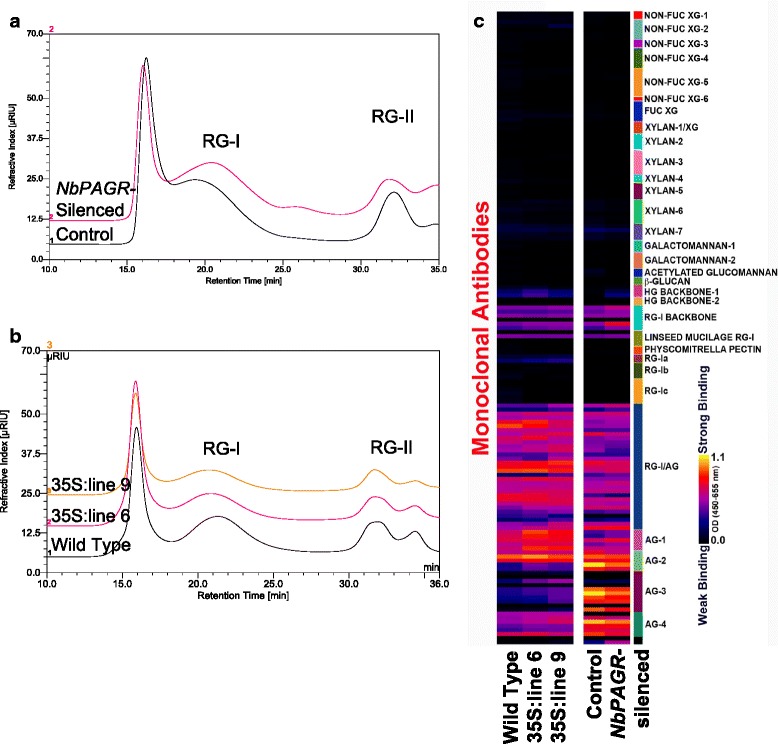
Characterization of RG-I in *PAGR-*overexpressing Arabidopsis lines and *NbPAGR*-silenced *N. benthamiana*. Size exclusion chromatography using a Superdex 200 column of RG-I from *NbPAGR*-silenced and control *N. benthamiana* cell walls (**a**) and RG-I from *35S::PAGR-YFP* lines and the wild type (**b**). RG-I from *NbPAGR-*silenced lines eluted later than the control indicating a decrease in the average molecular weight of RG-I domains. RG-I from PAGR-overexpressing Arabidopsis lines eluted slightly earlier than RG-I from the wild type. **c** ELISA analysis of size exclusion chromatography-purified RG-I with plant-glycan directed monoclonal antibodies

To further characterize the altered arabinogalactan content of cell walls from *NbPAGR-*silenced plants we analyzed the molecular mass and composition of RGI. RG-I can be solubilized from cell wall material along with RG-II by digestion with polygalacturonanase and pectin methylesterase [39]. RG-I released by this enzymatic treatment was separated from RG-II by size-exclusion chromatography on a Superdex 200 10/300 column (Fig. 7). RG-I from *NbPAGR*-silenced plants eluted later than RG-I from control plants. The average molecular mass of RG-I from silenced plants was calculated to be 91.2 kDa compared to 118.6 kDa in control plants when compared to the elution times of dextran molecular mass standards. RG-I fractions were collected following size exclusion chromatography and the monosaccharide composition of the collected material was analyzed. The arabinose and galactose content of RG-I was reduced from 21.9 % arabinose and 50.5 % galactose in control plants to 20.3 % arabinose and 44.4 % galactose in *NbPAGR-*silenced plants. Conversely the amount of RG-I backbone monosaccharides rhamnose and galacturonic acid was increased from 13.3 % rhamnose and 11.5 % galacturonic acid in the control to 16.6 % rhamnose and 15.6 % galacturonic acid in silenced plants (Table 4).

**Table 4:** The mol% monosaccharide composition of RG-I purified from *NbPAGR-*silenced and control *N. benthamiana* plants

	Control	*NbPAGR-*Silenced
Rha %	13.3	16.6
Ara %	21.9	20.3
Gal %	50.5	44.4
GalA %	11.5	15.6
GlcA %	1.9	1.8

We were then interested to determine if the decreased molecular mass of RG-I in *NbPAGR-*silenced plants was specifically due to reduced arabinogalactan content or if the RG-I backbone was truncated. From the approximate mass and monosaccharide composition of the isolated RG-I it is possible to estimate the approximate mass contribution of each monosaccharide comprising the RG-I molecule. First the approximate mass percentage (mass%) of each monosaccharide for the RG-I fractions was calculated by multiplying the mol% of each monosaccharide detected by its residue molecular mass (assuming an average of one glycosidic bond per residue) and then dividing by the total of these values for each sample. The mass% for each residue was then multiplied by the estimated total molecular mass of the RG-I fraction (Additional file 9: Table S1). This analysis showed that the amount of rhamnose and galacturonic acid residues in *NbPAGR-*silenced RG-I was nearly identical to that of the control with 14.9 kDa of rhamnose and 15.5 kDa of galacturonic acid residues in the control compared to 14.3 kDa of rhamnose and 16.1 kDa of galacturonic acid residues in RG-I from *NbPAGR-*silenced plants. The amounts of arabinose, galactose and glucuronic acid residues were decreased from 22.1 kDa of arabinose, 62.6 kDa of galactose and 2.6 kDa of glucuronic acid in control plants to 15.7 kDa of arabinose, 42.2 kDa of galactose and 1.8 kDa of glucuronic acid in RG-I from silenced plants. These data support that the reduced molecular mass of RG-I in *NbPAGR*-silenced plants is due to a reduction in arabinogalactan content of the polysaccharide, and is not due to a reduction in the degree of polymerization of the RG-I backbone. This analysis also allows us to estimate the number of residues making up each molecule of RG-I by dividing the calculated molecular mass contribution of each type of residue in the RG-I by the molecular mass of the monosaccharide residue (Table 5). This calculation yields a fairly consistent estimate of 90 to 100 rhamnose-galacturonic acid disaccharides comprising the RG-I backbone in both silenced and control plants.

**Table 5:** The estimated average number of monosaccharide residues making up each RG-I molecule in *NbPAGR-*Silenced and control *N. benthamiana* plants

	Control	*NbPAGR-*Silenced
Rha	102	98
Ara	168	119
Gal	386	260
GalA	88	91
GlcA	15	10

### *PAGR* overexpression increases rhamnogalacturonan-I arabinan content

RG-I from 35S::PAGR-YFP transgenic Arabidopsis seedlings was analyzed with the same method used for the *NbPAGR-*silenced *N. benthamiana* plants. RG-I was extracted from cell wall material enzymatically and then analyzed by size exclusion chromatography. In lines overexpressing *PAGR-YFP,* RG-I eluted at an earlier retention time of 20.9 and 20.8 min compared to 21.3 min in wild type seedlings (Fig. 7), corresponding to an increase in the molecular mass of RG-I from an average of 98.4 kDa to 106.8 kDa and 108.3 kDa when compared to the retention times of dextran molecular mass standards. We collected these RG-I fractions and analyzed the monosaccharide composition. In *PAGR* overexpressing plants we observed an increase in the mol% of arabinose from 24.1 % in the wild type to 30.2 % and 33.6 % in the 35S::PAGR-YFP lines (Table 6). The contribution of each monosaccharide to the total mass of the RG-I was calculated as for the RG-I from *NbPAGR*-silenced plants (Additional file 10: Table S2). This analysis showed an increase in the amount of arabinose residues from 20.2 kDa in the wild type to 27.9 and 31.8 kDa in the PAGR-overexpressing lines. There was also a decrease in the amount of glucuronic acid from 1.2 kDa in the wild type to 1.0 kDa and 0.8 kDa in the overexpressor lines as well as an increase in the amount of fucose from 1.0 kDa in the wild type to 1.3 and 1.4 kDa in the two *PAGR* overexpressing lines. The amount of rhamnose, galactose and galacturonic acid in RG-I from *PAGR* overexpressors was similar to that of the wild type. Next we calculated the approximate number of each monosaccharide residue making up the RG-I fractions. Again the number of rhamnose and galacturonic acid disaccharides making up the RG-I backbone was between 90 and 100 for both overexpressing and control plants (Table 7).

**Table 6:** The mol% monosaccharide composition of RG-I purified from 35S::PAGR-YFP lines and the wild type (Col0)

	Col-0	35S::PAGR-YFP line 6	35S-PAGR-YFP line 9
Fuc %	1.1	1.2	1.3
Rha %	13.8	12.3	13.1
Ara %	24.1	30.2	33.6
Gal %	42	39.8	37.3
Xyl/Man %	1.6	1.6	1.5
GalA %	16.3	14.3	12.6
GlcA %	1.1	0.8	0.6

**Table 7:** The estimated average number of monosaccharide residues making up each RG-I molecule in *35S::PAGR-YFP* and wild type (Col-0) plants

	Col-0	35S::PAGR-YFP line 6	35S-PAGR-YFP line 9
Fuc	7	8.7	9.5
Rha	88	86	94
Ara	153	211	241
Gal	266	278	267
Xyl/Man	10.3	10.8	10.9
GalA	104	100	90
GlcA	6.8	5.4	4.4

### *PAGR* affects the abundance of arabinogalactan and rhamnogalacturonan-I backbone epitopes in purified rhamnogalacturonan-I

In order to better understand the changes in RG-I composition induced by altered *PAGR* expression, the glycan epitope composition of RG-I samples purified by size exclusion chromatography from *NbPAGR-*silenced and control *Nicotiana benthamina* plants as well as PAGR-overexpressing and wild type Arabidopsis plants were analyzed by ELISA using a comprehensive set of plant glycan-directed antibodies [40] (Fig. 7c, Additional file 11: Table S3). These RG-I preparations were recognized nearly exclusively by antibodies directed at pectic backbone and arabinogalactan epitopes. Weak binding by antibodies against de-esterified homogalacturonan (HG) epitopes was observed in RG-I preparations from all plant lines, as expected given the methods used to generate the RG-I. RG-I from *NbPAGR*-silenced plants yielded stronger signals for antibodies that recognize un-branched RG-I backbone epitopes than did RG-I from the controls. RG-I from silenced plants also exhibited altered signal strength for antibodies binding arabinogalactan epitopes. RG-I from silenced plants produced reduced signals for some antibodies belonging to the AG-2, −3, and −4 groups of antibodies that recognize distinct arabinogalactan epitopes and subtly enhanced signals for many antibodies belonging to the AG-I group. RG-I from *PAGR-*overexpressing Arabidopsis plants showed no changes in signals for antibodies recognizing RG-I or HG backbone epitopes. Antibodies belonging to the AG-1 group produced stronger signals for RG-I from PAGR-overexpressing plants (lines 6 and 9) as did some antibodies in groups AG-3 and −4 that target other arabinogalactan epitopes. Thus, the ELISA analyses support the conclusion that *NbPAGR*-silencing affects branching of the RG-I backbone and the overall arabinogalactan composition of RG-I. ELISA results for RG-I from 35S::PAGR-YFP lines also support the conclusion that the arabinogalactan substitution of RG-I is altered in these lines.

### Glycosidic linkage analysis of rhamnogalacturonan-I fractions

RG-I possesses distinct types of arabinogalactan sidechains with different galactan backbone linkages; type-I arabinogalactan has a backbone of linear β-1,4-d-Gal*p* while type-II arabinogalactans have β-1,3-, β-1,6-and β-1,3,6-linked d-Gal*p.* To further investigate the observed changes in RG-I composition in silenced and overexpressing plants we conducted glycosidic linkage analysis by analyzing the partially methylated, partially acetylated alditol derivatives generated from purified RG-I fractions (Table 8). In RG-I from *NbPAGR-*silenced plants the molar percentage of 4-galactose was reduced from 47.9 % in the control to 42.0 %. The amount of 2-rhamnose in RG-I from *NbPAGR-*silenced plants was increased from 6.2 % in the control to 8.9 %. Notably the ratio of 2-rhamnose to 2,4-rhamnose was increased from 0.78:1 in the control to ~1:1 in *NbPAGR-*silenced plants, consistent with the immunological evidence that the RG-I backbone is less branched in *NbPAGR-*silenced plants. Small increases in the amounts of 3,5-arabinose and 6-galactose were also detected in RG-I from *NbPAGR-*silenced plants. In RG-I from *PAGR*-overexpressing Arabidopsis plants, significant increases in the amount of 3- and 6- linked galactose were detected in addition to increases in 3,6-substituted hexose (assumed to be galactose as no glucose was detected in HPAEC glycosyl composition analysis of these fractions). The amount of 4-galactose was reduced from 19.4 % in RG-I from the wild type to 16.0 % and 14.7 % in RG-I from the two *35S::PAGR-YFP* lines. Together these data support the conclusion that the amount of type-II arabinogalactan is increased in the *PAGR*-overexpressing lines and the amount of type-I arabinogalactan is slightly reduced. For linkage analysis a large amount of cell wall material was required and therefore Arabidopsis rosette leaves were used as a source material, not young liquid culture-grown seedlings in which the monosaccharide composition phenotype is most pronounced in the *35S::PAGR-YFP* lines. This is probably the reason that changes in RG-I arabinose content detected in RG-I purified from young seedlings were not observed in the older, soil-grown material used for linkage analysis.

**Table 8:** Linkage analysis of RG-I from *NbPAGR-*silenced *N. benthamiana* plants and *PAGR*-overexpressing Arabidopsis lines

	VIGS Control	*NbPAGR*-Silenced	Col-0	35S::PAGR-YFP line 6	35S-PAGR-YFP line 9
T-Ara	1.1 ± 0.4	1.0 ± 0.2	1.9 ± 0.5	1.4 ± 0.2	2.3 ± 0.6
2-Rha	6.2 ± 0.5	8.9 ± 0.1*	8.5 ± 1.0	7.1 ± 1.8	9.6 ± 1.1
3-Ara	0.7 ± 0.1	0.7 ± 0.1	1.3 ± 0.1	1.3 ± 0.1	1.2 ± 0.1
T-Gal	4.9 ± 0.4	6.7 ± 0.2	6.3 ± 0.3	6.0 ± 0.7	5.8 ± 0.5
5-Ara	8.4 ± 0.5	9.2 ± 0.7	16.5 ± 0.7	16.5 ± 0.6	16.0 ± 0.7
2,4-Rha	7.9 ± 0.1	8.8 ± 0.4	7.4 ± 1.2	7.6 ± 1.1	6.8 ± 0.4
3-Gal	4.4 ± 0.1	4.5 ± 0.3	3.5 ± 0.1	4.6 ± 0.2*	4.7 ± 0.2*
3,5-Ara	1.2 ± 0.1	1.4 ± 0.1*	6.4 ± 0.2	6.4 ± 0.5	5.9 ± 0.5
4-Gal	47.9 ± 0.4	42.0 ± 1.6*	19.4 ± 0.4	16.0 ± 0.6*	14.7 ± 0.6*
2,5-Ara	ND	ND	6.6 ± 0.4	6.1 ± 0.5	5.5 ± 0.5
6-Gal	2.5 ± 0.1	3.0 ± 0.2*	4.6 ± 0.3	5.6 ± 0.1*	6.3 ± 0.1*
3,4-Hex	1.7 ± 0.2	1.4 ± 0.1	8.9 ± 0.5	9.3 ± 1.3	8.0 ± 1.0
2,4-Hex	0.7 ± 0.1	0.6 ± 0.1	ND	ND	ND
4,6-Hex	1.6 ± 0.1	1.6 ± 0.2	1.8 ± 0.1	1.5 ± 0.1	1.3 ± 0.1
3,6-Hex	10.6 ± 0.2	10.4 ± 0.4	6.8 ± 0.3	10.7 ± 0.2*	11.7 ± 1.1*

Values are the molar percentage of total sugars detected. Data shown are averages ± standard deviation. Asterisks indicate significant difference from wild type or control plants (*p* < 0.01, t-test). (ND: not detected)

## Discussion

Results reported here indicate that *PAGR* positively affects the biosynthesis of type-II arabinogalactans when overexpressed in Arabidopsis while silencing of the *N. benthamiana* ortholog, *NbPAGR*, decreases substitution of RG-I with type-I arabinogalactans and reduces branching of the RG-I backbone. Proposing a simple hypothesis for the glycosyltransferase activity of PAGR to explain these discordant results is challenging. Many distinct arabinogalactan sidechain structures have been detected as branches on the RG-I backbone [41]. The pattern of these substitutions on the RG-I backbone has yet to be described.

In the biosynthesis of glucuronoxylan and xyloglucan, glycosyltransferases display strong acceptor substrate specificity. In the biosynthesis of glucuronoxylan, GUX1 shows a strong preference for the addition of glucuronic acid to evenly spaced xylan residues at intervals of around 8 residues while GUX2 adds glucuronic acid at intervals of between 5 and 7 residues with no preference for even or odd spacing [42]. The activity of these two glucuronosyltransferases creates distinct xylan domains within the same molecule with distinct substitution patterns. In the biosynthesis of xyloglucan, XLT2 and MUR3 specifically add galactose to distinct xylosyl residues in each xyloglucan subunit [43, 44]. We speculate that by altering the substitution of the RG-I backbone, PAGR may affect recognition of the RG-I backbone by enzymes synthesizing type-I and type-II arabiongalactans. Alternatively, PAGR might primarily affect substitution of the most abundant RG-I arabinogalactan sidechain. We observed that in *N. benthamiana*, where silencing of *NbPAGR* primarily affects substitution of RG-I with type-I arabinogalactans, these are the most abundant sidechains. In Arabidopsis RG-I, type-II arabinogalactans are significantly more abundant.

The overall biosynthetic process by which pectic polysaccharides are made continues to be debated [1, 16]. Two main models for the pectin biosynthetic process have been proposed; the consecutive GT model and the domain synthesis model. In the consecutive GT model GTs sequentially add sugars from nucleotide-sugars onto a growing pectin polysaccharide as they move through the Golgi apparatus. In the domain synthesis model oligo- or polysaccharide primers are synthesized from nucleotide sugars or lipid-linked sugars and elongated into pectic glycan domains. These pectic domains are then transferred onto a growing pectin molecule. Our data could also support PAGR being involved in a biosynthetic step prior to the transfer of glycosides or oligosaccharides onto a nascent RG-I and thus affect the production of more than one type of RG-I sidechain. This possible function for PAGR is similar to the hypothesized roles of Mannan Synthase-Related (MSR)-1 and −2 [24]. The MSRs are DUF246 domain containing, Golgi-localized GT-like proteins involved in mannan biosynthesis (see Additional file 2: Figure S2). In *msr1 msr2* double mutants mannosyl levels are reduced by approximately 50 % and mannan synthase activity is reduced. Wang et al., (2012) hypothesized that MSR proteins may function in the production of oligosaccharide primers for the synthesis of mannans or in stabilization of the Mannan Synthase.

That *PAGR* appears to be necessary for pollen tube growth but not pollen development suggests that *PAGR* has a role in the production of polysaccharides present in the pollen tube cell wall. Experiments with transgenic expression of enzymes capable of digesting pectic polysaccharides in potatoes have shown that these polysaccharides are essential for pollen viability [21]. A collapsed pollen phenotype and decreased male fertility were observed in potato lines expressing enzymes digesting the RG-I backbone or pectic arabinans. The presence of conserved *PAGR* orthologs in *Physcomitrella* and *Selaginella* support that its role has been conserved throughout the evolution of land plants. The involvement of PAGR in production of a critical polysaccharide structure, such as an early bond in RG-I arabinogalactan sidechains, could drive such a high level of conservation.

The morphological phenotypes induced by overexpression and silencing of PAGR and NbPAGR provide evidence supporting a role for pectic arabinogalactans in regulating the extensibility of plant cell walls. Pleiotropic morphological phenotypes are manifested only in specific organs of PAGR overexpressors, which suggests that critical properties of RG-I are substantially altered by PAGR overexpression only in specific tissues. The elasticity of cell walls in the shoot apical meristem is thought to play a key role in regulating organ formation [45]. Pectin methylesterification affects the elasticity of cell walls and is highly regulated in the shoot apical meristem, where regions of de-methyl-esterification underlie new lateral organ primordia [46]. Alteration of pectin methylesterification in the shoot apical meristem also affects the elasticity of cell walls and the production of new organs [47]. The altered phyllotaxy and fasciation of 35S::PAGR-YFP plants may be attributable to altered extensibility of cell walls in the shoot apical meristem. In *NbPAGR-*silenced plants decreased internode expansion and shortened roots are likely due to decreased cell wall extensibility. If PAGR has a role in the biosynthesis of RG-I arabinogalactan sidechains it is possible that both the biochemical and morphological phenotypes of PAGR overexpression may depend upon the degree of RG-I substitution normally present in a particular tissue. For example, in the young *PAGR*-overexpressing seedlings analyzed for RG-I monosaccharide composition, we observed a significant increase in RG-I arabinan content, while the similar linkage analysis of RG-I from rosette leaves did not show alterations in arabinan in *PAGR*-overexpressors.

## Conclusions

Together, the results presented here support that *PAGR* functions in the biosynthesis of RG-I arabinogalactans and illustrates the essential roles of these polysaccharides in vegetative and reproductive plant growth. More research is needed to understand the detailed structure of RG-I, particularly with respect to the pattern and nature of branching, and the biosynthetic process by which RG-I is produced in plants. Such research will better enable efforts to identify the specific biochemical role of PAGR in the biosynthesis of RG-I arabinogalactan sidechains.

## Methods

### Plant material

*Arabidopsis thaliana* Heyn. (L) accession Columbia-0 seeds were obtained from Lehle Seeds (Round Rock, Texas). Arabidopsis plants were grown under a 10-h photoperiod 22 °C with 90 μmol m^−2^ s^−1^ illumination during the day period. After 4 weeks, plants were transferred to a 16-h light photoperiod to induce flowering. *Nicotiana benthamiana* seeds were kindly provided by the Dinesh-Kumar Lab (Univerisity of California, Davis). *N. benthamiana* plants were grown under 16-h photoperiod at 25 °C, 60 % humidity with 200 μmol m^−2^s^−1^ illumination during the day period. Arabidopsis T-DNA lines CS836448 (*pagr-1*) and SALK_064738C (*pagr-2*) were acquired from the Arabidopsis Biological Resource Center (ABRC, Ohio State University). Genotyping of T-DNA lines was performed by PCR using genomic primers Cs836448F 5’-TCTTCCAGAGATAGAGCAGATGGCTG-3’ and Cs836448R 5’-TGCGCTTCTGCAAGGCGAGC-3’ for *pagr-1* and S_64738F 5’-TGGCGTCACTGGGTGCTCCT-3’ and S_64738R 5’-TCAGCCATCTGCTCTATCTCTGGAAG-3’ for *pagr-2.* T-DNAs were detected using the forward genomic primers and the appropriate left-border T-DNA primers pDAP101-Lb1 5’-GCCTTTTCAGAAATGGATAAATAGCCTTGCTTCC-3’ for *pagr-1* and LB1 5’-TGGTTCACGTAGTGGGCCATCG-3’ for *pagr-2. In vitro* pollen germination assays were performed as described [48]. Differential staining of aborted pollen grains was performed as described [26] and imaged using a Leica DMB4000B microscope.

### Virus Induced Gene Silencing (VIGS)

*NbPAGR-A* (*Niben101Scf07590g07020*) and *NbPAGR-B* (*Niben101Scf35628g00005*) were identified in the Sol Genomics Network Database [49] as reciprocal best BLAST hits for PAGR in the *N. benthamiana* genome. Alignment and phylogenetic analysis of the NbPAGR predicted amino acid sequences with the Arabidopsis DUF246 family proteins showed NbPAGR-A and -B to be the close homologs of Arabidopsis PAGR (Additional file 2: Figure S2). The *NbPAGR-A* sequence used to induce VIGS was amplified from total *N. benthamiana* cDNA using primers 5’-TTATCTAGACGATGACGATTACCGTGGCCGT-3’ and 5’-TTATCTAGAGCTGGTTTAGACCACCCTCAGCG-3’. Subsequently the fragment was cloned into the Xba1 site in pYL156 [34] i.e. pTRV2, to generate pYL156-NbPAGR. As a non-silencing control plasmid, pYL156 with a fragment of the *GUS* gene was used (pYC1) [50]. pYC1 and pYL156-NbPAGR were independently transformed into *Agrobacterium tumefaciens* strain GV3101. Virus-induced gene silencing was induced in 2–3 week old *N. benthamiana* plants according to standard protocols [34]. Tissue was collected 14 days post infection for all analyses. Sequence alignments were performed in Geneious 4.6.5 (Biomatters, New Zealand) Phylogenetic analyses were performed using *http://www.phylogeny.fr* [51].

### *PAGR* Overexpression and localization

The coding sequence of *At3g26370* (*PAGR*) was amplified from total Arabidopsis cDNA using Phusion High-Fidelity DNA polymerase (New England Biolabs) and the primers 5’-CACCATGGCAGAGTTACGGCACTCGAGCTCTCTC-3’ and 5’-TCCAGCTTTACACATGCATGGAGTGAGAGG-3’. The PCR product was cloned into pENTR-D-Topo (Invitrogen). A Gateway LR recombination reaction was performed according to the manufacturer’s protocol (Invitrogen) to transfer the coding sequence of *PAGR* into pGWB41 [52] to produce *35S::PAGR-YFP* for production of transgenic Arabidopsis plants. This construct was transformed into *A. tumefaciens* strain GV3101 and Arabidopsis plants of the Columbia-0 ecotype were transformed via the floral dip method [53]*.* For total cell wall and RG-I monosaccharide composition analysis of *35S::PAGR-YFP* lines, T3 seedlings were grown in liquid culture at 22 °C for 14 days. For localization studies, the coding sequence of PAGR was recombined into pGWB44 to product 35S::PAGR-CFP. *PAGR-CFP* was transiently co-expressed with α-mannosidase-1 [28] in 4-week-old *N. benthamiana* leaves following described procedures [54] except that 100 mM 2-(N-morpholino)ethanesulfonic acid, 100 mM MgCl_2_, 10 μM acetosyringone was used as the infiltration medium. Expression in *N. benthamiana* epidermal cells was imaged using a Zeiss 710 confocal laser-scanning microscope (Carl Zeiss).

### Immunoblotting

Total protein was extracted from 1 week old Arabidopsis seedlings by grinding in 100 mM Tris pH 7.5, 1 mM EDTA, 1 % (v/v) Triton X-100, 10 % (v/v) glycerol, 1 mM phenylmethylsulfonyl fluoride. Cellular debris was pelleted by centrifugation at 16,000 x *g* for 15 min at 4 °C. Extracted protein was quantified by Bradford Assay [55]. For electrophoresis, 40 μg of protein was separated by SDS-PAGE, electrotransferred onto a PVDF membrane and incubated with AttB2 site ‘universal’ primary antibody and anti-rabbit secondary antibody as described by [56], except that 5 % w/v BSA (Sigma) in TBS-T was used as a blocking agent and membranes were incubated with universal antibody at a 1:3000 dilution.

### Quantitative RT-PCR

RNA was extracted from Arabidopsis and *N. benthamiana* tissues using the RNEasy plant mini kit (Qiagen). For cloning purposes RNA was extracted from *N. benthamiana* tissues using Trizol Reagent (Invitrogen). cDNA was synthesized with Superscript-III reverse transcriptase (Invitrogen). Quantitative RT-PCR was performed on a StepOne-Plus Real-Time PCR system (Applied BioSystems) using Syber-Select Real-Time PCR reagents (Invitrogen). *PAGR* was detected using primer At3g26370 q1F 5’-GAGGTCGTCGCAGATCTTCAGGTTCATGT-3’ and At3g26370 q1R 5’-GGCTCCCACTGTTCTTCTTCATCAGGCTT-3’. MONENSIN SENSITIVITY1, At2g28390, a gene with exceptional transcript-level stability [57], was analyzed as a reference gene using primers 5’-AACTCTATGCAGCATTTGATCCACT-3’ and 5’-TGATTGCATATCTTTATCGCCATC-3’. Data were analyzed using the comparative Ct method. For quantitative real-time RT-PCR analysis of *NbPAGR* silencing, data were analyzed using the geometric mean of three reference genes as the common reference as described [58]. For *NbPAGR-A* and *–B,* primers *NbPAGR-A*qF 5’-CTACGCCACTCAAGCTCGATCGGAAA-3’, *NbPAGR-A*qR 5’-GCCACGGTAATCGTCATCGTCATCGTCAA-3’, *NbPAGR-B*qF 5’-CTACGCCACTCAAGCTCGATCGGAAG-3’ and *NbPAGR-B*qR 5’-GCCACGGTAATCGTCATCGTCATCGTCAT-3’ were used. Elongation Factor 1A, Actin-2 and Ubiquitin 3 were used as reference genes using Elongation Factor 1A primers NbEF1qF 5’-AGGGTCCAACCCTCCTTGAGGC-3’ and NbEF1qR 5’-GCCCCTTTGGCTGGGTCGTC-3’; Actin-2 primers ACT2F 5’-TTGAGACTTTTAATACCCCAGC-3’ and ACT2R 5’-AACATGTAACCACGCTCGGTAA-3’ and Ubiquitin-3 primers UBQ3F 5’-GCCGATTACAACATCCAGAAGG-3’ and UBQ3R 5’-TGAAGTACAGCGAGCTTAACC-3’.

### Cell wall isolation and monosaccharide composition analysis

Alcohol-insoluble residue (AIR) was prepared as described by [12]. Lyophilized AIR was hydrolyzed in 2 M trifluoroacetic acid at 120 °C for 1 h and analyzed by high-performance anion exchange chromatography (HPAEC) as described by [59]. Glucose was not determined for samples of total cell wall material due to the presence of residual starch. Sequential extraction of AIR was performed essentially as described [60] with the exception that samples from *PAGR*-overexpressors were not extracted with 1 M KOH prior to the extraction with 4 M KOH.

### Endo-β-1,4-galactanase digestion

Cell wall preparations from VIGS plants were further analyzed by digestion with endo-β-1,4-galactanase from *Aspergillus niger* purified to a single band on a silver-stained gel (Megazyme, product code E-EGALN). AIR (2 mg) was dissolved in 0.1 mL of 1 M KOH and adjusted to pH 4.7 with 2 mL of 100 mM acetic acid. Galactanase was added and samples were incubated at 40 °C for 1 h at 40 °C. After incubation, cold ethanol with 10 mM EDTA was added to a final concentration of 70 % (v/v) and the sample was centrifuged for 5 min at 14,000 x *g* at 4 °C. The supernatant and pellet were separated, hydrolyzed and analyzed by HPAEC as described above.

### Rhamnogalacturonan-I purification

Rhamnogalacturonan-I was isolated essentially as described by [39]. Briefly, 15 mg of AIR was digested overnight with 3U of pectin methyl-esterase (Novoshape Pure PME, Novozymes) and 20U of endopolygalacturonanase M2 (Megazyme, product code E-PGALUSP) at 37 °C in 1 ml of 50 mM ammonium oxalate (pH 5.0). Following digestion, insoluble material was removed by centrifugation followed by filtration through a 0.2 μm spin filter. Oligosaccharides and the digestion buffer were removed by washing of the solubilized polysaccharides on a 10 kDa Molecular Weight Cutoff spin filter (Amicon) with sterile water. Samples were eluted from the spin concentrators in water and separated by size-exclusion chromatography in 50 mM ammonium formate (pH5.0) on a Superdex 200 10/300GL column (GE Healthcare Bio-Sciences, http://www.gelifesciences.com/) at a flow rate of 0.5 ml/min. Elution of polysaccharides from the column was monitored with a Shodex RI-101 refractive index detector (Shodex, http://www.shodex.com). Fractions were collected manually, lyophilized, hydrolyzed and analyzed by HPAEC as described above. Estimates of the MW of RG-I were made with reference to the retention times of Dextran MW standards (Sigma-Aldrich). The relative mass percentage of each monosaccharide in the RG-I fractions was determined by first calculating the mass ratio of each monosaccharide by dividing the product of the mol% and the molar mass of each monosaccharide by the sum of the products of the molar mass and mol% of each monosaccharide. We then multiplied the monosaccharide mass ratios by the estimated molecular weight of the RG-I fraction.

### ELISA screening of purified rhamnogalacturonan-I preparations

Purified RG-I samples were dissolved in water and were coated onto ELISA plates [384 well clear flat bottom polystyrene high bind microplate (product no. 3700), Corning Life Sciences] on an equal weight (gravimetric) per well basis (0.5 μg/well). The samples were then subjected to ELISA screening with a comprehensive suite of cell wall glycan-directed monoclonal antibodies essentially as described earlier [40, 61]. The ELISA screening assays were done using an Robotic System (Thermo Scientific) comprising an Orbitor RS Robotic Arm (Thermo Scientific) accessing the following components: Carousel plate storage/incubation (Thermo Scientific), EON Plate Reader (Biotek), EL406 ELISA Plate Washer (Biotek), Multiflo Dispenser (Biotek) and Precision XS Fluid Dispenser (Biotek). The whole system is operated by the laboratory automation software, Momentum 3.2.7 (Thermo Scientific). Water was used as the blanks and these background values were subtracted from the sample ELISA responses. The ELISA assays were done in technical duplicates and data represent the average of the replicates. Cell wall glycan-directed antibodies were were obtained from laboratory stocks (CCRC, JIM and MAC series) at the Complex Carbohydrate Research Center (available through CarboSource Services; http://www.carbosource.net) or through BioSupplies (Australia) (BG1, LAMP).

### Rhamnogalacturonan-I linkage analysis

Glycosidic linkage analysis of RG-I was performed by GC/MS analysis of their partially methylated alditol acetates [62]. Purified RG-I samples were per-*O*-methylated using liquid NaOH in dimethyl sulfoxide and further derivatized to their corresponding partially methylated alditol acetates by trifluoroacetic acid hydrolysis, reduction and per-*O*-acetylation. The derivatives were separated using a gas chromatograph (Agilent 7890A, Agilent Technologies, www.agilent.com) equipped with a Supelco SP2380 column (Sigma-Aldrich) and a mass spectrometer (Agilent 5975C) using a temperature gradient as described [60]. Eluted compounds were identified based on their retention time compared to standards and their ion fragmentation patterns.

## Ethics approval and consent to participate

Not applicable.

## Consent for publication

Not applicable.

## Availability of data and materials

The data sets supporting the results of this article are included within the article and its additional files. Nucleotide sequences and biological materials including Arabidopsis seeds, plasmids and bacterial strains created through this work are available at (https://registry.jbei.org). Data supporting phylogenetic analyses presented in this study are available at (http://purl.org/phylo/treebase/phylows/study/TB2:S19106).

## Additional files

Additional file 1: Figure S1. Multiple amino acid sequence alignment of the *Arabidopsis thaliana, Nicotiana benthamiana, Selaginella moellendorfii* and *Physcomitrella patens* orthologs of *PAGR.* * identical amino acids, : conserved substitutions, . semi-conserved substitutions. (PPTX 36 kb) Additional file 2: Figure S2. Phylogenetic analysis of the DUF246 containing proteins in Arabidopsis, *Selaginella moellendorffi* and *Physcomitrella patens.* After manual curation of sequences, phylogenetic analysis was performed using MUSCLE alignment, phyML and statistical analysis using SH-like approximate likelihood-test. The tree was generated using *http://www.phylogeny.fr/* [49]. (PNG 187 kb) Additional file 3: Figure S3. Tissue specific microarray data heat map depicting the relative expression level of *PAGR* in various tissues throughout growth and development. Publicly available data was retrieved and displayed using the Arabidopsis eFP browser (http://bar.utoronto.ca/efp/cgi-bin/efpWeb.cgi) [23]. (PPTX 235 kb) Additional file 4: Figure S4. Root phenotypes of *NbPAGR-*silenced and TRV-infected unsilenced control plants. (A) Roots of *NbPAGR-*silenced plants are shorter than those of control plants and were discolored. (B) Roots of *NbPAGR-*silenced and control plants were measured and were found to be significantly shorter in s * *p* < 0.05, *n* = 4, brackets indicate one standard deviation (B). Discoloration of *NbPAGR*-silenced roots (D) compared to control roots (C). (PPTX 4221 kb) Additional file 5: Figure S5. Additional images of in vitro pollen germination assays showing reduced germination rates in pollen from *pagr-1* and *pagr-2* heterozygous plants compared to the wild type. Scale bars are 100 μm. (PPTX 1073 kb) Additional file 6: Figure S6. Phylogenetic analysis of the DUF246 containing proteins in Arabidopsis and the PAGR orthologs identified in *Nicotiana benthamiana, Selaginella moellendorffi* and *Physcomitrella patens.* After manual curation of sequences, phylogenetic analysis was performed using MUSCLE alignment, phyML and statistical analysis using SH-like approximate likelihood-test. The tree was generated using *http://www.phylogeny.fr/* [49]. (PNG 23 kb) Additional file 7: Figure S7. Sequential extraction of cell wall material from *NbPAGR-*silenced *Nicotiana benthamiana* plants. Cell wall material from *NbPAGR-*silenced and control plants was sequentially extracted with CDTA, sodium carbonate, 1 M KOH and 4 M KOH. The monosaccharide composition of the extracted and residual materials was then analyzed. Cell wall polysaccharides with reduced galactan and increased glucuronic acid content in *PAGR-*silenced plants were extracted with the CDTA and sodium carbonate fractions. *: *p* < 0.001, t-test, *n* = 4. (PPTX 47 kb) Additional file 8: Figure S8. Sequential extraction of cell walls from *35S:PAGR-YFP* expressing Arabidopsis seedlings. Cell wall material from was sequentially extracted with CDTA, sodium carbonate, and 4 M KOH. The monosaccharide composition of the extracted and residual materials was then analyzed. Cell wall polysaccharides with increased arabinan content were extracted in the CDTA fraction. *: *p* < 0.001, t-test, *n* = 4. (PPTX 45 kb) Additional file 9: Table S1. The approximate mass (kDa) of each monosaccharide residue in RG-I from *NbPAGR-*silenced and control *N. benthamiana* plants. (DOCX 39 kb) Additional file 10: Table S2. The approximate mass (kDa) of each monosaccharide residue in RG-I purified from *35S::PAGR-YFP* and wild type (Col-0) plants. (DOCX 44 kb) Additional file 11: Table S3. ELISA absorbance values for RG-I fractions from *NbPAGR-*silenced and virus-infected, control *N. benthamiana* plants as well as 35S::PAGR-YFP and wild type Arabidopsis plants probed with a diverse array of plant cell wall glycan-directed monoclonal antibodies. (XLSX 21 kb)
