# Supplementary material for: A DUF-246 family glycosyltransferase-like gene affects male fertility and the biosynthesis of pectic arabinogalactans
Source: BMC Plant Biol. 2016 Apr 18;16:90. doi: 10.1186/s12870-016-0780-x (PMC4836069; doi:10.1186/s12870-016-0780-x)
Supplement: Additional file 1: Figure S1. — Multiple amino acid sequence alignment of the Arabidopsis thaliana, Nicotiana benthamiana, Selaginella moellendorfii and Physcomitrella patens orthologs of PAGR. * identical amino acids, : conserved substitutions, . semi-conserved substitutions. (PPTX 36 kb) [file 12870_2016_780_MOESM1_ESM.pptx]

## Slide 1
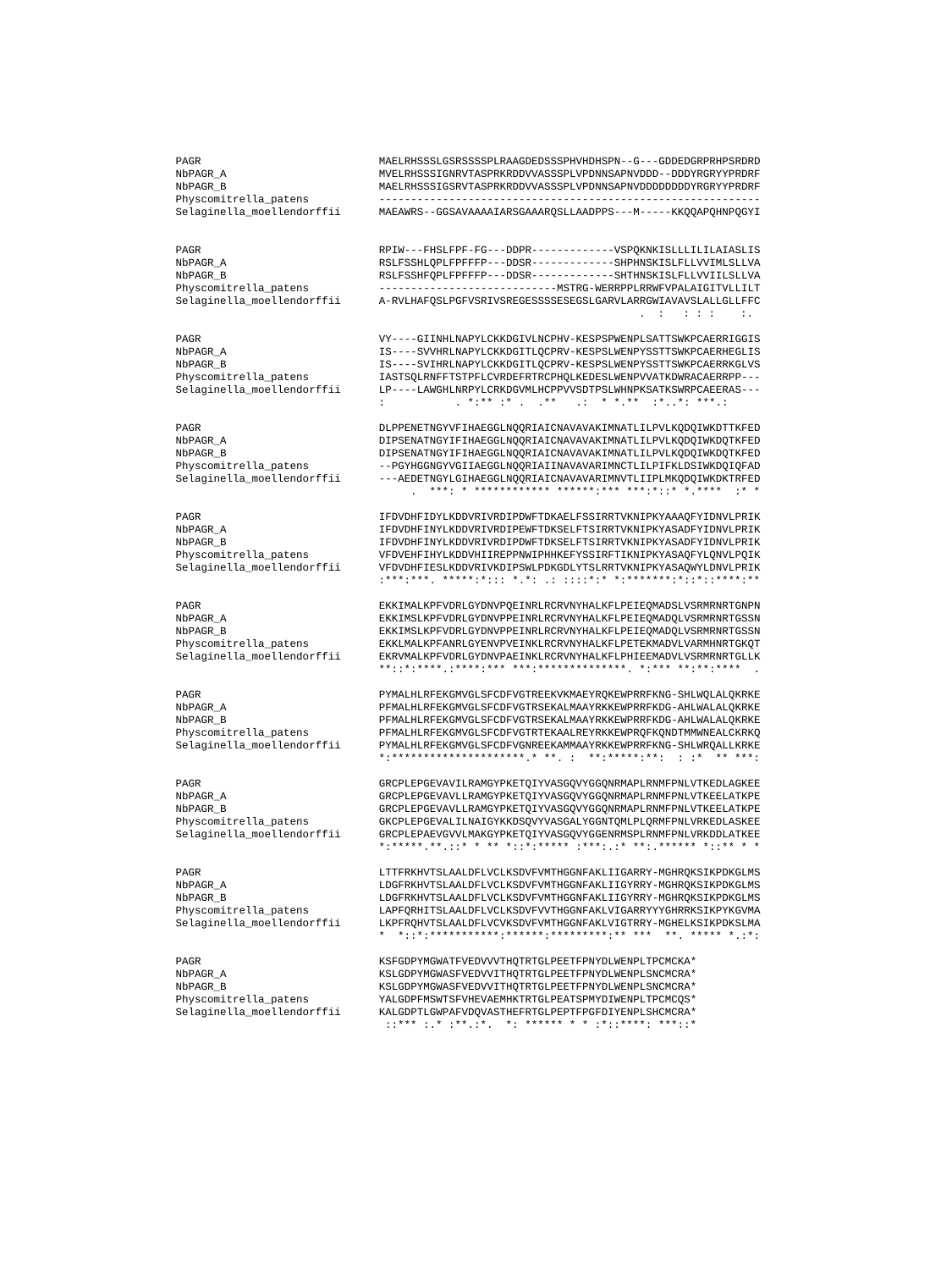

PAGR MAELRHSSSLGSRSSSSPLRAAGDEDSSSPHVHDHSPN--G---GDDEDGRPRHPSRDRD
NbPAGR_A MVELRHSSSIGNRVTASPRKRDDVVASSSPLVPDNNSAPNVDDD--DDDYRGRYYPRDRF
NbPAGR_B MAELRHSSSIGSRVTASPRKRDDVVASSSPLVPDNNSAPNVDDDDDDDDYRGRYYPRDRF
Physcomitrella_patens ------------------------------------------------------------
Selaginella_moellendorffii MAEAWRS--GGSAVAAAAIARSGAAARQSLLAADPPS---M-----KKQQAPQHNPQGYI
PAGR RPIW---FHSLFPF-FG---DDPR-------------VSPQKNKISLLLILILAIASLIS
NbPAGR_A RSLFSSHLQPLFPFFFP---DDSR-------------SHPHNSKISLFLLVVIMLSLLVA
NbPAGR_B RSLFSSHFQPLFPFFFP---DDSR-------------SHTHNSKISLFLLVVIILSLLVA
Physcomitrella_patens ----------------------------MSTRG-WERRPPLRRWFVPALAIGITVLLILT
Selaginella_moellendorffii A-RVLHAFQSLPGFVSRIVSREGESSSSESEGSLGARVLARRGWIAVAVSLALLGLLFFC
 . : : : : :.
PAGR VY----GIINHLNAPYLCKKDGIVLNCPHV-KESPSPWENPLSATTSWKPCAERRIGGIS
NbPAGR_A IS----SVVHRLNAPYLCKKDGITLQCPRV-KESPSLWENPYSSTTSWKPCAERHEGLIS
NbPAGR_B IS----SVIHRLNAPYLCKKDGITLQCPRV-KESPSLWENPYSSTTSWKPCAERRKGLVS
Physcomitrella_patens IASTSQLRNFFTSTPFLCVRDEFRTRCPHQLKEDESLWENPVVATKDWRACAERRPP---
Selaginella_moellendorffii LP----LAWGHLNRPYLCRKDGVMLHCPPVVSDTPSLWHNPKSATKSWRPCAEERAS---
 : . *:** :* . .** .: * *.** :*..*: ***.:
PAGR DLPPENETNGYVFIHAEGGLNQQRIAICNAVAVAKIMNATLILPVLKQDQIWKDTTKFED
NbPAGR_A DIPSENATNGYIFIHAEGGLNQQRIAICNAVAVAKIMNATLILPVLKQDQIWKDQTKFED
NbPAGR_B DIPSENATNGYIFIHAEGGLNQQRIAICNAVAVAKIMNATLILPVLKQDQIWKDQTKFED
Physcomitrella_patens --PGYHGGNGYVGIIAEGGLNQQRIAIINAVAVARIMNCTLILPIFKLDSIWKDQIQFAD
Selaginella_moellendorffii ---AEDETNGYLGIHAEGGLNQQRIAICNAVAVARIMNVTLIIPLMKQDQIWKDKTRFED
 . ***: * ************ ******:*** ***:*::* *.**** :* *
PAGR IFDVDHFIDYLKDDVRIVRDIPDWFTDKAELFSSIRRTVKNIPKYAAAQFYIDNVLPRIK
NbPAGR_A IFDVDHFINYLKDDVRIVRDIPEWFTDKSELFTSIRRTVKNIPKYASADFYIDNVLPRIK
NbPAGR_B IFDVDHFINYLKDDVRIVRDIPDWFTDKSELFTSIRRTVKNIPKYASADFYIDNVLPRIK
Physcomitrella_patens VFDVEHFIHYLKDDVHIIREPPNWIPHHKEFYSSIRFTIKNIPKYASAQFYLQNVLPQIK
Selaginella_moellendorffii VFDVDHFIESLKDDVRIVKDIPSWLPDKGDLYTSLRRTVKNIPKYASAQWYLDNVLPRIK
 :***:***. *****:*::: *.*: .: ::::*:* *:*******:*::*::****:**
PAGR EKKIMALKPFVDRLGYDNVPQEINRLRCRVNYHALKFLPEIEQMADSLVSRMRNRTGNPN
NbPAGR_A EKKIMSLKPFVDRLGYDNVPPEINRLRCRVNYHALKFLPEIEQMADQLVSRMRNRTGSSN
NbPAGR_B EKKIMSLKPFVDRLGYDNVPPEINRLRCRVNYHALKFLPEIEQMADQLVSRMRNRTGSSN
Physcomitrella_patens EKKLMALKPFANRLGYENVPVEINKLRCRVNYHALKFLPETEKMADVLVARMHNRTGKQT
Selaginella_moellendorffii EKRVMALKPFVDRLGYDNVPAEINKLRCRVNYHALKFLPHIEEMADVLVSRMRNRTGLLK
 **::*:****.:****:*** ***:**************. *:*** **:**:**** .
PAGR PYMALHLRFEKGMVGLSFCDFVGTREEKVKMAEYRQKEWPRRFKNG-SHLWQLALQKRKE
NbPAGR_A PFMALHLRFEKGMVGLSFCDFVGTRSEKALMAAYRKKEWPRRFKDG-AHLWALALQKRKE
NbPAGR_B PFMALHLRFEKGMVGLSFCDFVGTRSEKALMAAYRKKEWPRRFKDG-AHLWALALQKRKE
Physcomitrella_patens PFMALHLRFEKGMVGLSFCDFVGTRTEKAALREYRKKEWPRQFKQNDTMMWNEALCKRKQ
Selaginella_moellendorffii PYMALHLRFEKGMVGLSFCDFVGNREEKAMMAAYRKKEWPRRFKNG-SHLWRQALLKRKE
 *:*********************.* **. : **:*****:**: : :* ** ***:
PAGR GRCPLEPGEVAVILRAMGYPKETQIYVASGQVYGGQNRMAPLRNMFPNLVTKEDLAGKEE
NbPAGR_A GRCPLEPGEVAVLLRAMGYPKETQIYVASGQVYGGQNRMAPLRNMFPNLVTKEELATKPE
NbPAGR_B GRCPLEPGEVAVLLRAMGYPKETQIYVASGQVYGGQNRMAPLRNMFPNLVTKEELATKPE
Physcomitrella_patens GKCPLEPGEVALILNAIGYKKDSQVYVASGALYGGNTQMLPLQRMFPNLVRKEDLASKEE
Selaginella_moellendorffii GRCPLEPAEVGVVLMAKGYPKETQIYVASGQVYGGENRMSPLRNMFPNLVRKDDLATKEE
 *:*****.**.::* * ** *::*:***** :***:.:* **:.****** *::** * *
PAGR LTTFRKHVTSLAALDFLVCLKSDVFVMTHGGNFAKLIIGARRY-MGHRQKSIKPDKGLMS
NbPAGR_A LDGFRKHVTSLAALDFLVCLKSDVFVMTHGGNFAKLIIGYRRY-MGHRQKSIKPDKGLMS
NbPAGR_B LDGFRKHVTSLAALDFLVCLKSDVFVMTHGGNFAKLIIGYRRY-MGHRQKSIKPDKGLMS
Physcomitrella_patens LAPFQRHITSLAALDFLVCLKSDVFVVTHGGNFAKLVIGARRYYYGHRRKSIKPYKGVMA
Selaginella_moellendorffii LKPFRQHVTSLAALDFLVCVKSDVFVMTHGGNFAKLVIGTRRY-MGHELKSIKPDKSLMA
 * *::*:***********:******:*********:** *** **. ***** *.:*:
PAGR KSFGDPYMGWATFVEDVVVTHQTRTGLPEETFPNYDLWENPLTPCMCKA*
NbPAGR_A KSLGDPYMGWASFVEDVVITHQTRTGLPEETFPNYDLWENPLSNCMCRA*
NbPAGR_B KSLGDPYMGWASFVEDVVITHQTRTGLPEETFPNYDLWENPLSNCMCRA*
Physcomitrella_patens YALGDPFMSWTSFVHEVAEMHKTRTGLPEATSPMYDIWENPLTPCMCQS*
Selaginella_moellendorffii KALGDPTLGWPAFVDQVASTHEFRTGLPEPTFPGFDIYENPLSHCMCRA*
 ::*** :.* :**.:*. *: ****** * * :*::****: ***::*
