# Supplementary figures and images for: A DUF-246 family glycosyltransferase-like gene affects male fertility and the biosynthesis of pectic arabinogalactans
Source: BMC Plant Biol. 2016 Apr 18;16:90. doi: 10.1186/s12870-016-0780-x (PMC4836069; doi:10.1186/s12870-016-0780-x)

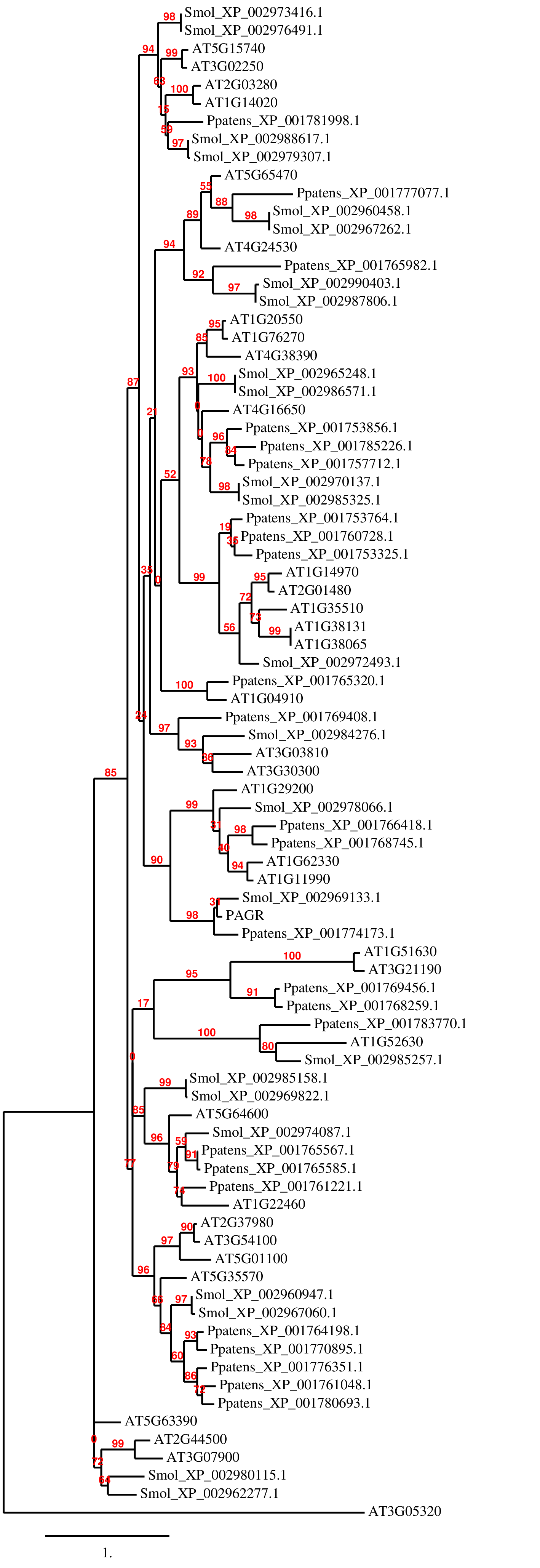

Supplement: Additional file 2: Figure S2. — Phylogenetic analysis of the DUF246 containing proteins in Arabidopsis, Selaginella moellendorffi and Physcomitrella patens. After manual curation of sequences, phylogenetic analysis was performed using MUSCLE alignment, phyML and statistical analysis using SH-like approximate likelihood-test. The tree was generated using http://www.phylogeny.fr/ [49]. (PNG 187 kb) [file 12870_2016_780_MOESM2_ESM.png]

## Slide 1
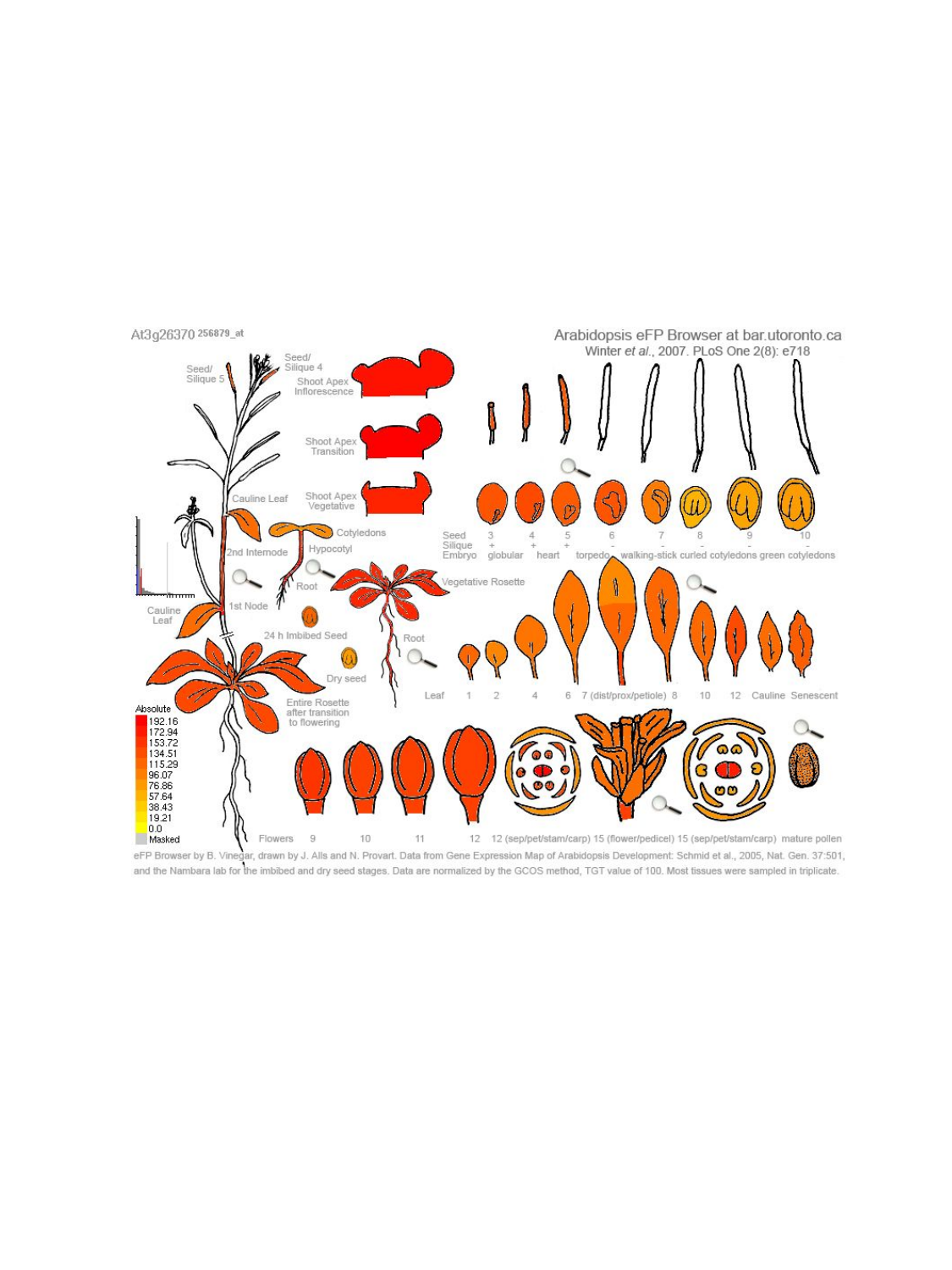

Supplement: Additional file 3: Figure S3. — Tissue specific microarray data heat map depicting the relative expression level of PAGR in various tissues throughout growth and development. Publicly available data was retrieved and displayed using the Arabidopsis eFP browser (http://bar.utoronto.ca/efp/cgi-bin/efpWeb.cgi) [23]. (PPTX 235 kb) [file 12870_2016_780_MOESM3_ESM.pptx]

## Slide 1
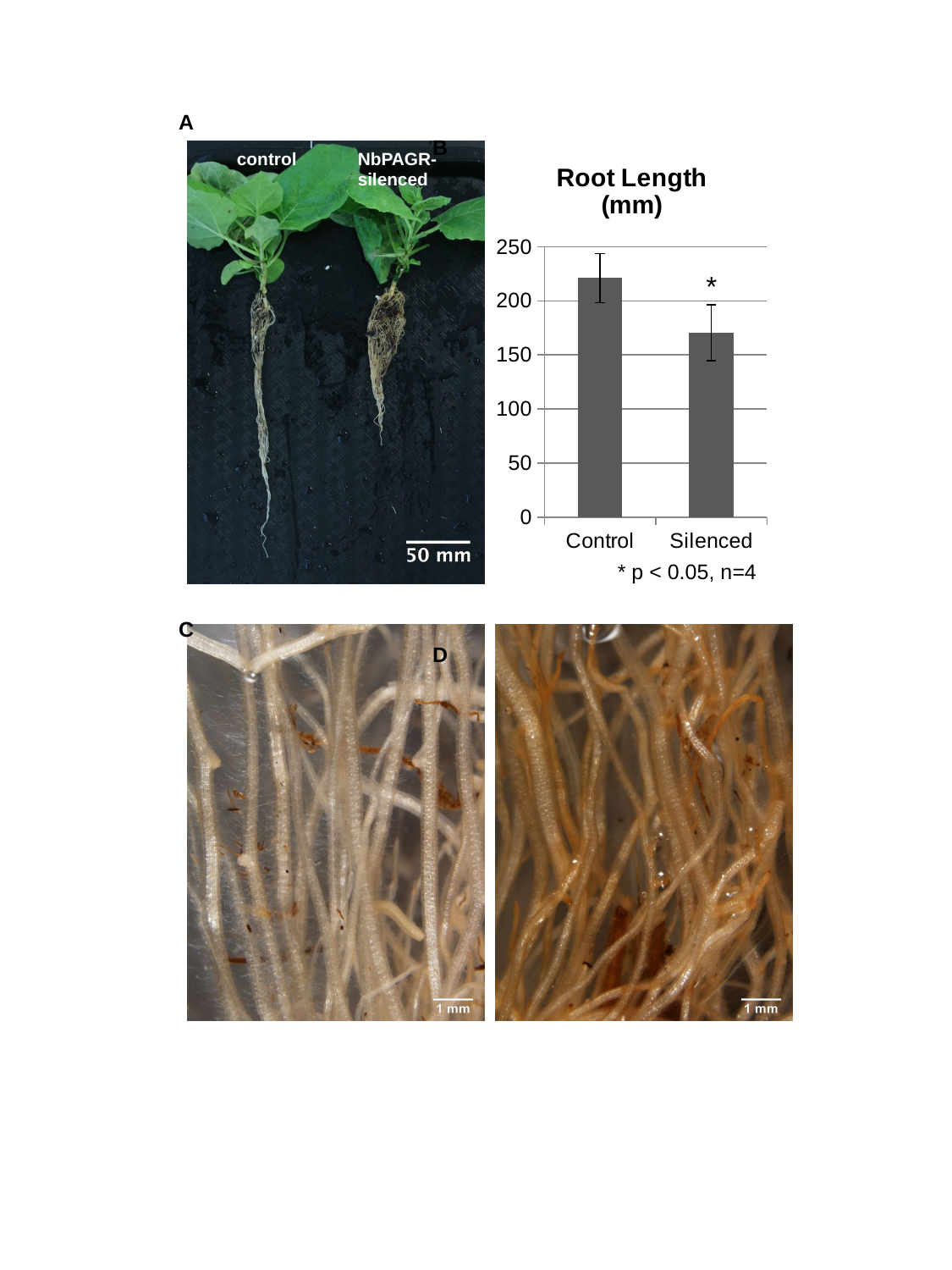

A					B
C					D
### Chart: Root Length (mm)
| Category | |
|---|---|
| Control | 221.0 |
| Silenced | 170.5 |
control
NbPAGR-silenced
* p < 0.05, n=4

Supplement: Additional file 4: Figure S4. — Root phenotypes of NbPAGR-silenced and TRV-infected unsilenced control plants. (A) Roots of NbPAGR-silenced plants are shorter than those of control plants and were discolored. (B) Roots of NbPAGR-silenced and control plants were measured and were found to be significantly shorter in s * p < 0.05, n = 4, brackets indicate one standard deviation (B). Discoloration of NbPAGR-silenced roots (D) compared to control roots (C). (PPTX 4221 kb) [file 12870_2016_780_MOESM4_ESM.pptx]

## Slide 1
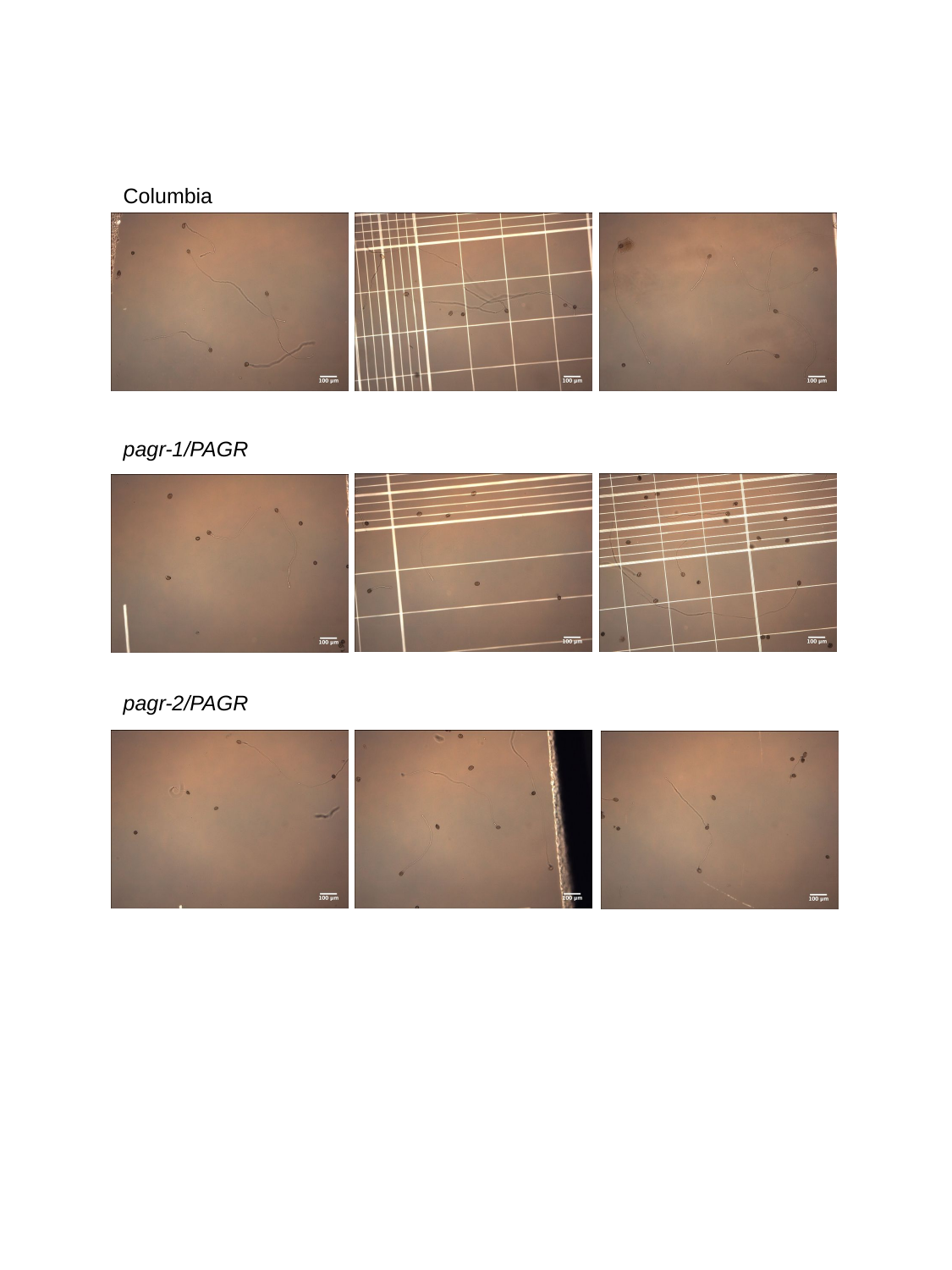

Columbia
pagr-1/PAGR
pagr-2/PAGR

Supplement: Additional file 5: Figure S5. — Additional images of in vitro pollen germination assays showing reduced germination rates in pollen from pagr-1 and pagr-2 heterozygous plants compared to the wild type. Scale bars are 100 μm. (PPTX 1073 kb) [file 12870_2016_780_MOESM5_ESM.pptx]

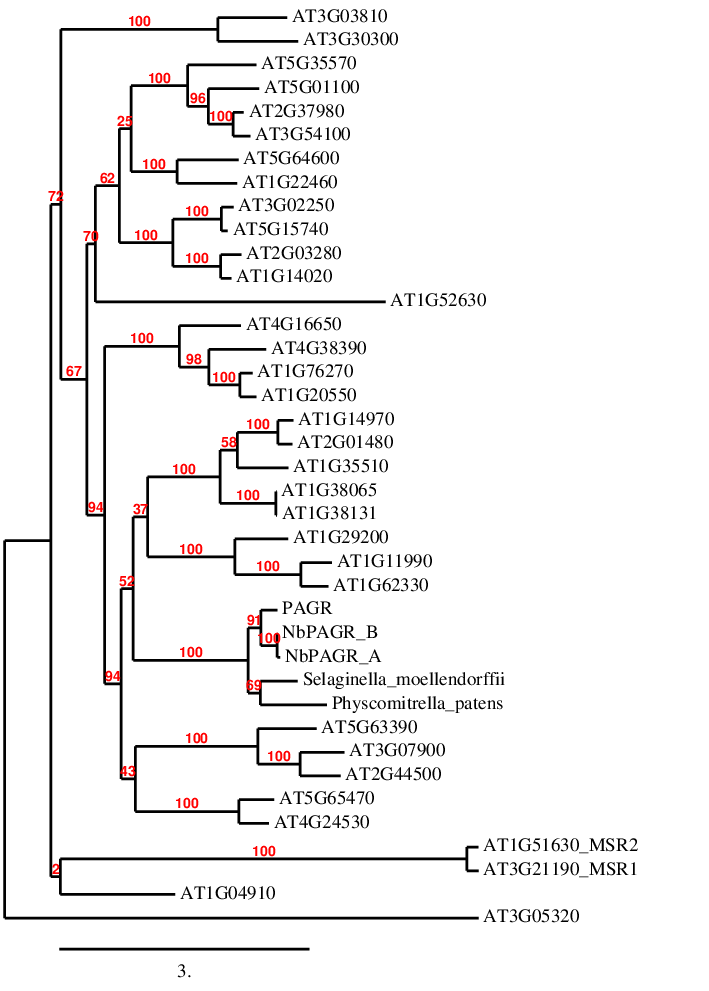

Supplement: Additional file 6: Figure S6. — Phylogenetic analysis of the DUF246 containing proteins in Arabidopsis and the PAGR orthologs identified in Nicotiana benthamiana, Selaginella moellendorffi and Physcomitrella patens. After manual curation of sequences, phylogenetic analysis was performed using MUSCLE alignment, phyML and statistical analysis using SH-like approximate likelihood-test. The tree was generated using http://www.phylogeny.fr/ [49]. (PNG 23 kb) [file 12870_2016_780_MOESM6_ESM.png]
