# Supplementary material for: A DUF-246 family glycosyltransferase-like gene affects male fertility and the biosynthesis of pectic arabinogalactans
Source: BMC Plant Biol. 2016 Apr 18;16:90. doi: 10.1186/s12870-016-0780-x (PMC4836069; doi:10.1186/s12870-016-0780-x)
Supplement: Additional file 7: Figure S7. — Sequential extraction of cell wall material from NbPAGR-silenced Nicotiana benthamiana plants. Cell wall material from NbPAGR-silenced and control plants was sequentially extracted with CDTA, sodium carbonate, 1 M KOH and 4 M KOH. The monosaccharide composition of the extracted and residual materials was then analyzed. Cell wall polysaccharides with reduced galactan and increased glucuronic acid content in PAGR-silenced plants were extracted with the CDTA and sodium carbonate fractions. *: p < 0.001, t-test, n = 4. (PPTX 47 kb) [file 12870_2016_780_MOESM7_ESM.pptx]

## Slide 1
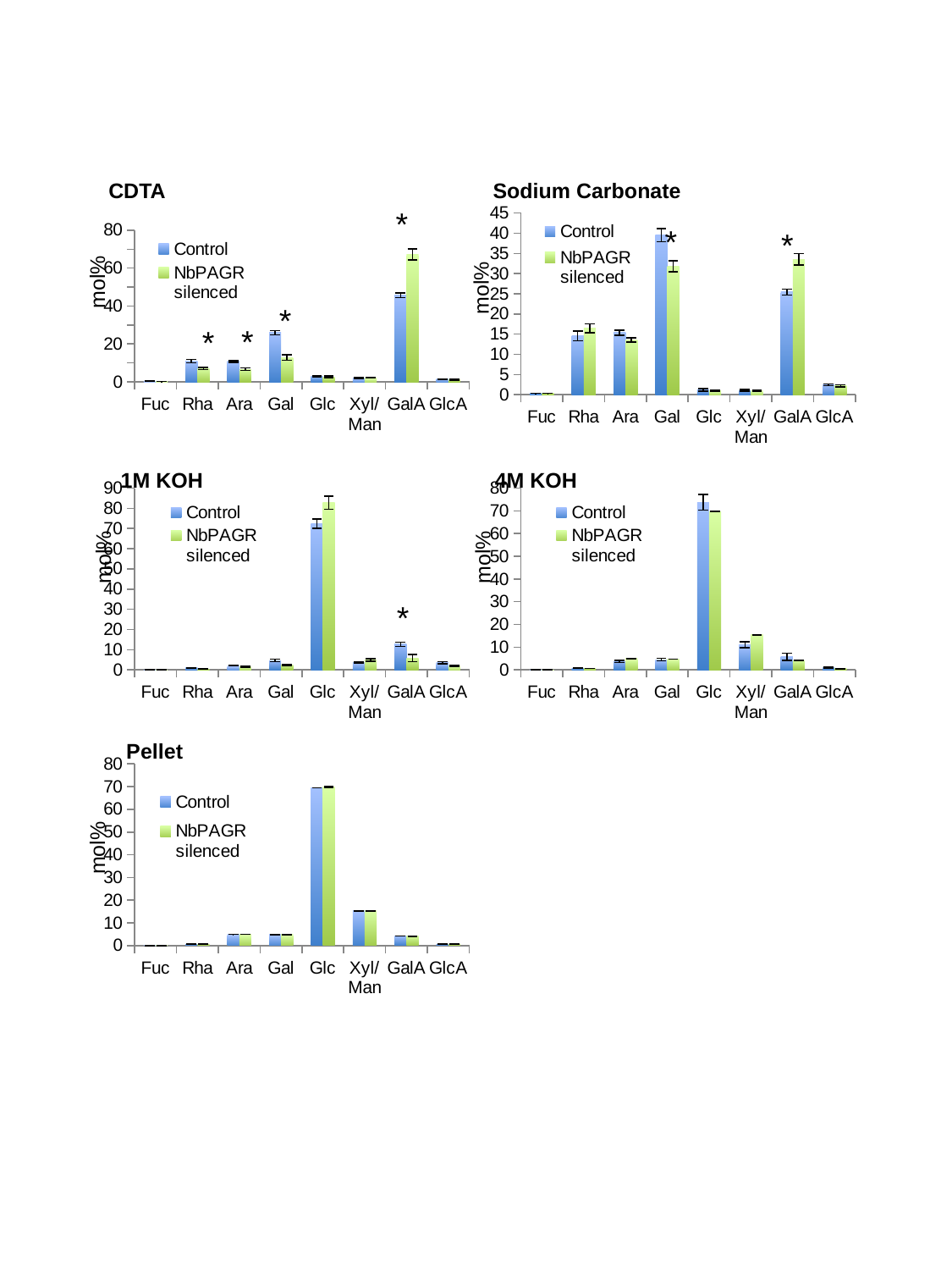

Sodium Carbonate
CDTA
*
### Chart
| Category | Control | NbPAGR silenced |
|---|---|---|
| Fuc | 0.325205178033592 | 0.358305769794681 |
| Rha | 14.55908495097006 | 16.45151375145794 |
| Ara | 15.38667458399131 | 13.59661037884924 |
| Gal | 39.56905679430812 | 31.84072412023778 |
| Glc | 1.20967570725304 | 1.060492379924822 |
| Xyl/Man | 1.086085532184105 | 0.952107488017012 |
| GalA | 25.41667446824048 | 33.5082449601115 |
| GlcA | 2.447542785019275 | 2.232001151607031 |
### Chart
| Category | Control | NbPAGR silenced |
|---|---|---|
| Fuc | 0.363772259344337 | 0.213228570107608 |
| Rha | 10.97589231950268 | 6.977702200839791 |
| Ara | 10.74749928503351 | 6.71438244893289 |
| Gal | 25.99210212148785 | 12.88262981698171 |
| Glc | 2.849254452126485 | 2.683120597554053 |
| Xyl/Man | 1.933815702958389 | 2.131924079858524 |
| GalA | 45.67788249185777 | 67.31055894223678 |
| GlcA | 1.550724432525067 | 1.086453343488643 |*
*
mol%
mol%
*
*
*
1M KOH
4M KOH
### Chart
| Category | Control | NbPAGR silenced |
|---|---|---|
| Fuc | 0.0 | 0.0 |
| Rha | 0.968610072952857 | 0.454266818565337 |
| Ara | 2.123346314069124 | 1.660108708564628 |
| Gal | 4.628000458969105 | 2.387633899244896 |
| Glc | 72.3807955360665 | 82.779667777737 |
| Xyl/Man | 3.726163745991323 | 4.853463011828841 |
| GalA | 12.68084781484801 | 5.868385935572315 |
| GlcA | 3.492236057102685 | 1.996473848486965 |
### Chart
| Category | Control | NbPAGR silenced |
|---|---|---|
| Fuc | 0.0 | 0.0 |
| Rha | 0.75981376063574 | 0.606734279863362 |
| Ara | 3.616851915643238 | 4.95086130669337 |
| Gal | 4.455353515692033 | 4.760055857929402 |
| Glc | 73.7959210936373 | 69.6086948212282 |
| Xyl/Man | 10.99740186969746 | 15.35808156647866 |
| GalA | 5.688854558923293 | 4.153231551862357 |
| GlcA | 0.914404381027858 | 0.562340615944625 |mol%
mol%
*
Pellet
### Chart
| Category | Control | NbPAGR silenced |
|---|---|---|
| Fuc | 0.0 | 0.0 |
| Rha | 0.601060030616344 | 0.590903305947743 |
| Ara | 4.965689346211885 | 5.01566503058913 |
| Gal | 4.811180230234537 | 4.89934119243197 |
| Glc | 69.50610179831187 | 69.66349435045787 |
| Xyl/Man | 15.30369808421232 | 15.24350897801893 |
| GalA | 4.225689919737034 | 4.17513937479757 |
| GlcA | 0.586580590676001 | 0.549263690342063 |mol%
