# Supplementary material for: A DUF-246 family glycosyltransferase-like gene affects male fertility and the biosynthesis of pectic arabinogalactans
Source: BMC Plant Biol. 2016 Apr 18;16:90. doi: 10.1186/s12870-016-0780-x (PMC4836069; doi:10.1186/s12870-016-0780-x)
Supplement: Additional file 8: Figure S8. — Sequential extraction of cell walls from 35S:PAGR-YFP expressing Arabidopsis seedlings. Cell wall material from was sequentially extracted with CDTA, sodium carbonate, and 4 M KOH. The monosaccharide composition of the extracted and residual materials was then analyzed. Cell wall polysaccharides with increased arabinan content were extracted in the CDTA fraction. *: p < 0.001, t-test, n = 4. (PPTX 45 kb) [file 12870_2016_780_MOESM8_ESM.pptx]

## Slide 1
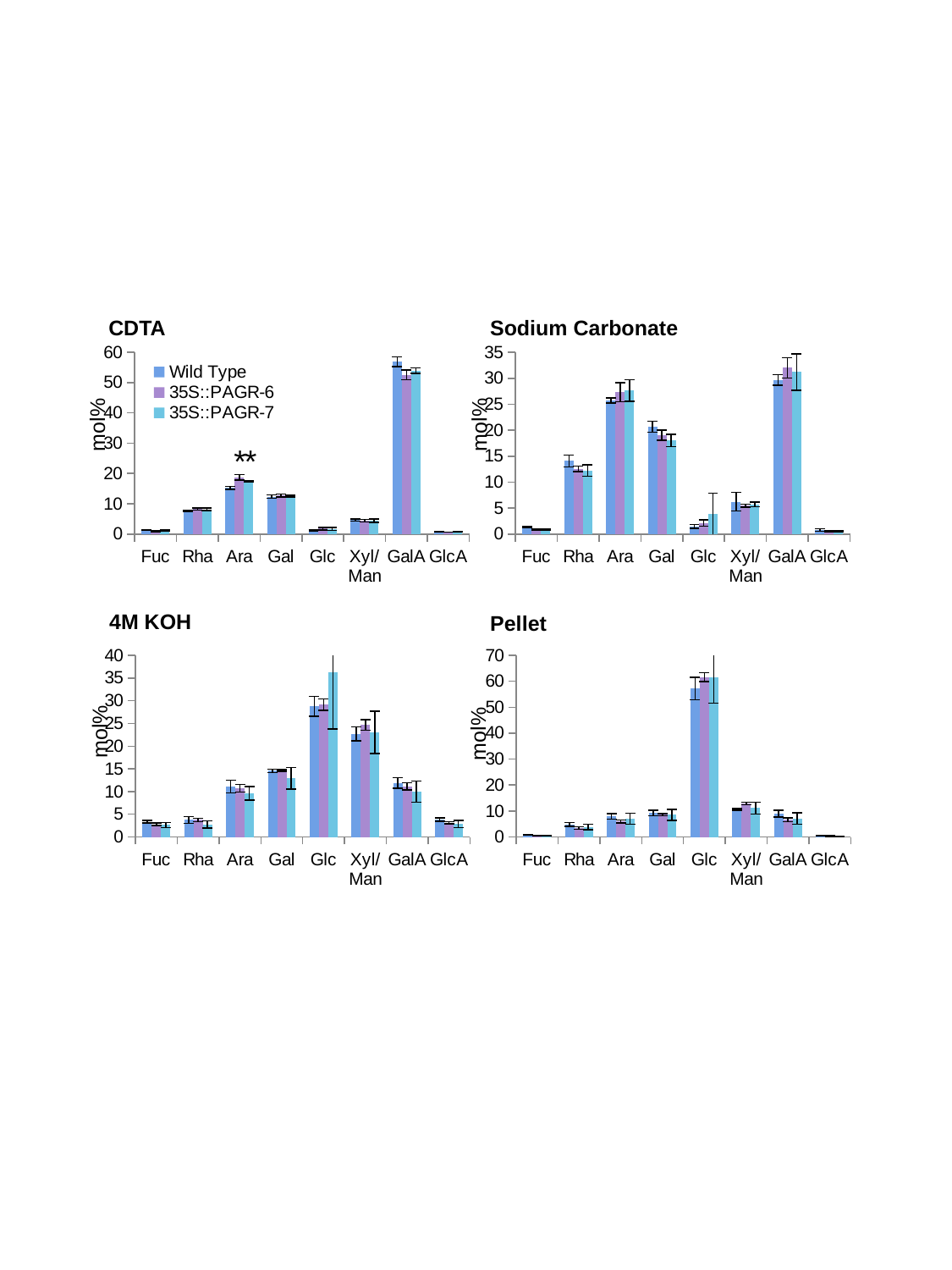

Sodium Carbonate
CDTA
### Chart
| Category | Wild Type | 35S::PAGR-6 | 35S::PAGR-7 |
|---|---|---|---|
| Fuc | 1.333143066891851 | 0.979128771336632 | 1.110666370970901 |
| Rha | 7.592482315876564 | 8.237466241873413 | 8.123969419300725 |
| Ara | 15.23697162482329 | 18.72607541065856 | 17.48860586727456 |
| Gal | 12.32223973732002 | 12.73244860444555 | 12.5122456782711 |
| Glc | 1.15771823179891 | 1.752575224913544 | 1.682346850908058 |
| Xyl/Man | 4.646625647946055 | 4.414876953246431 | 4.390739621264172 |
| GalA | 56.90168249721189 | 52.52260768032967 | 53.96278411778732 |
| GlcA | 0.809136878131405 | 0.634821113196182 | 0.728642074223159 |
### Chart
| Category | Wild Type | 35S::PAGR-6 | 35S::PAGR-7 |
|---|---|---|---|
| Fuc | 1.384944198499637 | 0.91144792139775 | 0.798446333713671 |
| Rha | 14.0703686982027 | 12.53844687367001 | 12.21230444938736 |
| Ara | 25.740585414564 | 27.33622082253012 | 27.62894420086388 |
| Gal | 20.66466983955693 | 19.05749888392385 | 17.99513798527235 |
| Glc | 1.486396415175762 | 2.103277332449226 | 3.908818139741382 |
| Xyl/Man | 6.21901681553489 | 5.481000080920498 | 5.721449696702011 |
| GalA | 29.6899842554986 | 32.0206431263049 | 31.20458134358675 |
| GlcA | 0.744034362967482 | 0.551464958803642 | 0.530317850732578 |mol%
mol%
*
*
4M KOH
Pellet
### Chart
| Category | Col | 35S::PAGR-6 | 35S::PAGR-7 |
|---|---|---|---|
| Fuc | 3.363104131810686 | 2.792711351115306 | 2.630889011587112 |
| Rha | 3.7418248044105 | 3.752947725217647 | 2.766945878975911 |
| Ara | 11.13250881219683 | 10.74281969313298 | 9.58712121477722 |
| Gal | 14.57327572808146 | 14.64322486450388 | 12.9145507849268 |
| Glc | 28.7419687619104 | 29.14313399992978 | 36.19275573475884 |
| Xyl/Man | 22.69394967875382 | 24.62472625759985 | 23.04304157939142 |
| GalA | 11.89235434315888 | 11.13742675347519 | 9.987963931632173 |
| GlcA | 3.861013739677438 | 3.163009355025356 | 2.87673186395054 |
### Chart
| Category | Wild Type | 35S::PAGR-6 | 35S::PAGR-7 |
|---|---|---|---|
| Fuc | 0.87522189002549 | 0.566752015432921 | 0.585625809883111 |
| Rha | 4.89238266352701 | 3.417955803316981 | 3.839403755821761 |
| Ara | 7.908923146973041 | 6.012845040069437 | 7.066261856092709 |
| Gal | 9.17510634493857 | 8.571715265683704 | 8.494209987003858 |
| Glc | 57.17217668476451 | 61.60408728635352 | 61.45525458228231 |
| Xyl/Man | 10.59865079282933 | 12.90423704452044 | 11.14026471713203 |
| GalA | 8.900304152456687 | 6.587094373975016 | 7.092954366448977 |
| GlcA | 0.477234324485363 | 0.335313170647974 | 0.326024925335239 |mol%
mol%
