# Supplementary material for: A DUF-246 family glycosyltransferase-like gene affects male fertility and the biosynthesis of pectic arabinogalactans
Source: BMC Plant Biol. 2016 Apr 18;16:90. doi: 10.1186/s12870-016-0780-x (PMC4836069; doi:10.1186/s12870-016-0780-x)
Supplement: Additional file 9: Table S1. — The approximate mass (kDa) of each monosaccharide residue in RG-I from NbPAGR-silenced and control N. benthamiana plants. (DOCX 39 kb) [file 12870_2016_780_MOESM9_ESM.docx]

Table S1: The approximate mass (kDa) of each monosaccharide residue in RG-I from *NbPAGR-*silenced and control *N. benthamiana* plants.

|  | **Control** | ***NbPAGR-*Silenced** |
| --- | --- | --- |
| Est. Total MW | 118.7 | 91.2 |
| Rha | 14.9 | 14.3 |
| Ara | 22.1 | 15.7 |
| Gal | 62.6 | 42.2 |
| GalA | 15.5 | 16.1 |
| GlcA | 2.6 | 1.8 |
| RG-I backbone (Rha + GalA) | 30.3 | 30.4 |
