# Supplementary material for: A DUF-246 family glycosyltransferase-like gene affects male fertility and the biosynthesis of pectic arabinogalactans
Source: BMC Plant Biol. 2016 Apr 18;16:90. doi: 10.1186/s12870-016-0780-x (PMC4836069; doi:10.1186/s12870-016-0780-x)
Supplement: Additional file 10: Table S2. — The approximate mass (kDa) of each monosaccharide residue in RG-I purified from 35S::PAGR-YFP and wild type (Col-0) plants. (DOCX 44 kb) [file 12870_2016_780_MOESM10_ESM.docx]

Table S2: The approximate mass (kDa) of each monosaccharide residue in RG-I purified from *35S::PAGR-YFP* and wild type (Col-0) plants.

|  | **Col-0** | **35S::PAGR-YFP line 6** | **35S-PAGR-YFP line 9** |
| --- | --- | --- | --- |
| Est. Total MW | 98.1 | 106.8 | 108.3 |
| Fuc | 1 | 1.3 | 1.4 |
| Rha | 12.8 | 12.5 | 13.7 |
| Ara | 20.2 | 27.9 | 31.8 |
| Gal | 43.2 | 45.1 | 43.4 |
| Xyl/Man | 1.4 | 1.4 | 1.4 |
| GalA | 18.3 | 17.6 | 15.8 |
| GlcA | 1.2 | 1 | 0.8 |
| RG1 backbone (Rha + GalA) | 31.1 | 30.1 | 29.5 |
